# Supplementary material for: Preconception health and care policies, strategies and guidelines in the UK and Ireland: a scoping review protocol
Source: BMJ Open. 2023 May 5;13(5):e067822. doi: 10.1136/bmjopen-2022-067822 (PMC10163469; doi:10.1136/bmjopen-2022-067822)
Supplement: Supplementary data [file bmjopen-2022-067822supp001.pdf]

Appendices:

Appendix I: Preconception risk factors

**Social**		
Adverse childhood experiences<br>Belonging to an ethnic minority<br>Deprivation<br>Domestic abuse (physical and emotional)	Environmental tobacco smoke and second-hand smoke exposure<br>Exposure to toxic or hazardous substances<br>Financial insecurity or unemployment<br>Inadequate support from family or social network	Low educational attainment<br>Migration status<br>Unsuitable access to quality healthcare and dentalcare<br>Unsuitable housing
**Behavioural**		
Alcohol misuse<br>Eating disorders<br>Inadequate dietary habits	Lack of family planning/pregnancy intention<br>Physical inactivity or sedentary behaviours	Smoking<br>Substance misuse<br>Use of teratogenic medications
**Biomedical**		
Fertility issues<br>Folic acid or other vitamin deficiency<br>General health status<br>Genetic risks<br>Infections (e.g., sexually transmitted diseases)	Low immunisation levels<br>Mental health conditions (e.g., anxiety, depression)<br>Parental age<br>Parental obesity or overweight, parental underweight	Physical health conditions (e.g., diabetes mellitus, epilepsy, endometriosis, chronic hypertension)<br>Previous pregnancy and obstetric complications (e.g., previous foetal death, miscarriage, stillbirth, preterm birth)

References for the table: [2,5,6]

Appendix II: Glossary of terms.

**Term**	**Definition**
Preconception health	The overall health of non-pregnant individuals of childbearing age[1].
Preconception care	The “services, interventions, support and advice” aimed at optimising pregnancy planning and fitness for pregnancy[2].
Interconception	Period between the end of a pregnancy and the start of the next[29].

Cassinelli EH, *et al. BMJ Open* 2023; 13:e067822. doi: 10.1136/bmjopen-2022-067822

Appendix III: Inclusion and exclusion criteria for a scoping review investigating strategies, policies, services, guidelines, frameworks and recommendations that address preconception health and care for adults in the UK and Ireland.

**Inclusion**	**Exclusion**
The resource is one of the following: strategies, policies, guidelines, frameworks and recommendations, including technical or research reports from government agencies, registered charities or scientific research groups, documents outlining interventions or initiatives from public bodies, articles and guidelines issued by government agencies, Royal Colleges and professional bodies, e-learning resources, leaflets and educational booklets. If the resource is a service, intervention or initiative it will be included only if pertinent to Northern Ireland.	The resource is one of the following: journal articles, preprints (journal articles not yet peer-reviewed or published), working papers from research groups, visual or audio content (or reviews of visual or audio content), academic letters or commentaries, calls for participants, presentations and doctoral dissertations. If the resource is a service, intervention or initiative it will be excluded if not pertinent to Northern Ireland.
The resource addresses preconception health and care.	The resource does not address preconception health and care. This includes if the resource explicitly addresses sexual health, interconception health and care or pregnancy.
The resource is relevant to the UK or Ireland.	The resource is not relevant to the UK or Ireland.
The resource was published, reviewed or updated during or after January 2011.	The resource was published, reviewed or updated before January 2011.
The resource was published in English.	The resource was published in any language other than English.
The resource presents unique insights and does not simply report, cite or signpost other included resources.	The resource mentions preconception health and care only by reporting, citing or signposting included resources, and therefore duplicates findings of an earlier version of the resource.
The resource is currently available.	The resource is not currently available (e.g., guideline being updated).

Cassinelli EH, *et al. BMJ Open* 2023; 13:e067822. doi: 10.1136/bmjopen-2022-067822

Appendix VI: Search strategy

List of searches conducted on Advanced Google Search for Northern Ireland.

	**Search terms**
1	~preconception AND "Northern Ireland" OR NI
2	~preconception AND "Northern Ireland" AND policy OR service OR intervention OR guideline OR strategy OR initiative
3	"preconception health" OR "preconception care" AND "Northern Ireland" OR NI
4	~pre-pregnancy AND "Northern Ireland" OR NI AND policy OR service OR intervention OR guideline OR strategy OR initiative
5	~preconception AND "Northern Ireland" OR NI AND ~charity
6	~preconception AND "Health and Social Care Trust" OR HSCT
7	"pregnancy planning" OR "planning a baby" OR "preparation for pregnancy" OR "preparation for parenthood" OR "pregnancy intention" OR "expecting mothers" OR "expectant mothers" OR "conceive" OR "future pregnancy" AND "Northern Ireland"
8	"reproductive health" OR "sexual health" AND "Northern Ireland" AND policy OR service OR intervention OR guideline OR strategy OR initiative

Results limited based on language (English) and country (United Kingdom).

Appendix V: Additional intended sources and websites

Source	Website	Context
National Institute for Health Research	https://www.nihr.ac.uk/	UK
British National Health Service (NHS)	https://www.nhs.uk/	UK
Royal College of Obstetricians and Gynaecologists	https://www.rcog.org.uk/	UK
Tommy's	https://www.tommys.org/	England, Scotland, Wales
Office for Health Improvement & Disparities	https://www.gov.uk/government/organisations/office-for-health-improvement-and-disparities	England
Public Health Wales	https://phw.nhs.wales/	Wales
Public Health Scotland	https://www.publichealthscotland.scot/	Scotland
NHS Inform	https://www.nhsinform.scot/	Scotland
Health Well	https://healthwell.eani.org.uk	Northern Ireland
Department of Health (Northern Ireland)	https://www.health-ni.gov.uk/	Northern Ireland
Sexual Health NI	https://www.sexualhealthni.info/	Northern Ireland
NI DIRECT	https://www.nidirect.gov.uk/	Northern Ireland
Public Health Agency	https://www.publichealth.hscni.net/	Northern Ireland
Health Service Executive IE	https://www.hse.ie/eng/	Ireland
Health Information and Quality Authority	https://www.hiqa.ie/areas-we-work/health-information/data-collections/all-ireland-public-health-repository	Ireland

Appendix VI: Example of completed data extraction instrument.

Reference #	Reviewed by	Title & url	Resource format	Source	Date accessed	Date published/last updated or modified	Duration, when applicable	Context	Notes/Short description	Key themes
63	EHC, LM	Hypertension in pregnancy - Quality standard [QS35] > https://www.nice.org.uk/guidance/qs35	Guideline	NICE	02-May-22	2019	NA	UK	"Women of childbearing potential with treated hypertension are given information annually about safe antihypertensive treatment during pregnancy." "Information can be provided to women who may become pregnant about safe antihypertensive treatment during pregnancy as part of an annual review of hypertension care. Women should be informed about potential risks, including the risk of congenital abnormalities, linked to particular antihypertensive drugs. This should enable women to arrange a discussion with the healthcare professional responsible for managing their hypertension about alternative antihypertensive treatments if they are planning pregnancy or become pregnant." (excerpts)	Blood pressure, hypertension, long-term conditions, Preconception advice

Appendix VII: Revised data extraction instrument.

**Data to be extracted**	**Notes to reviewer**
Reference #	
Reviewed by	
Title	
Resource format	e.g., report, intervention, strategy, policy
Source	e.g., Google Advanced Search, NICE, OpenAire
Date accessed	
Year of publication/Year of most recent update.	
Duration	When applicable, the duration of the strategy, policy or intervention will be reported
Participants*	Target population
Context	Country
Target audience*	e.g., healthcare professionals, policymakers
Overarching aim*	e.g., educate, increase awareness
Key notes on topics/content	

* Added following piloting phase.
