# Supplementary material for: Preconception health and care policies, strategies and guidelines in the UK and Ireland: a scoping review protocol
Source: BMJ Open. 2023 May 5;13(5):e067822. doi: 10.1136/bmjopen-2022-067822 (PMC10163469; doi:10.1136/bmjopen-2022-067822)
Supplement: Author's manuscript [file bmjopen-2022-067822.draft_revisions.pdf]

BMJ Open is committed to open peer review. As part of this commitment we make the peer review history of every article we publish publicly available.

When an article is published we post the peer reviewers' comments and the authors' responses online. We also post the versions of the paper that were used during peer review. These are the versions that the peer review comments apply to.

The versions of the paper that follow are the versions that were submitted during the peer review process. They are not the versions of record or the final published versions. They should not be cited or distributed as the published version of this manuscript.

BMJ Open is an open access journal and the full, final, typeset and author-corrected version of record of the manuscript is available on our site with no access controls, subscription charges or pay-per-view fees (http://bmjopen.bmj.com).

If you have any questions on BMJ Open's open peer review process please email info.bmjopen@bmj.com

BMJ Open

Preconception health and care policies, strategies and guidelines in the UK and Ireland: A scoping review protocol

Journal:	*BMJ Open*
Manuscript ID	bmjopen-2022-067822
Article Type:	Protocol
Date Submitted by the Author:	15-Sep-2022
Complete List of Authors:	Cassinelli, Emma ; Queen's University Belfast,<br>McKinley, Michelle C.; Queen's University Belfast<br>Kent, Lisa; Queen's University Belfast, Centre for Public Health<br>Eastwood, Kelly-Ann; University Hospitals Bristol and Weston NHS Foundation Trust; Queen's University Belfast<br>Schoenaker , Danielle; University of Southampton,<br>Trew, David; Patient and Public Involvement and Engagement<br>Stoikidou, Theano ; Patient and Public Involvement and Engagement<br>McGowan, Laura; Queen's University Belfast
Keywords:	Prenatal diagnosis < OBSTETRICS, PUBLIC HEALTH, Maternal medicine < OBSTETRICS, Reproductive medicine < GYNAECOLOGY, Protocols & guidelines < HEALTH SERVICES ADMINISTRATION & MANAGEMENT

SCHOLARONE™
Manuscripts

Title: Preconception health and care policies, strategies and guidelines in the UK and Ireland: A scoping review protocol

Authors: Emma Hope Cassinelli[1], Prof Michelle McKinley[1], Dr Lisa Kent[1], Dr Kelly-Ann Eastwood [1,2], Dr Danielle Schoenaker[3], David Trew[4], Theano Stoikidou[4], Dr Laura McGowan[1]

1. Centre for Public Health (Institute for Global Food Security), School of Medicine, Dentistry and Biomedical Sciences, Queen's University Belfast, Belfast (United Kingdom).
2. University Hospitals Bristol NHS Foundation Trust, Bristol (United Kingdom).
3. NIHR Southampton Biomedical Research Centre, University of Southampton and University Hospital Southampton NHS Foundation Trust, Southampton (United Kingdom).
4 Patient and Public Involvement and Engagement "Healthy Reproductive Years" Panel

Corresponding author: Dr Laura McGowan, Lecturer in Nutrition and Behaviour Change.
Postal address: Institute for Global Food Security (Centre for Public Health), Queen's University Belfast, Institute of Clinical Sciences, Block A. Grosvenor Road, Belfast, BT12 6BJ, United Kingdom.
Email address: laura.mcgowan@qub.ac.uk

For peer review only

For peer review only - http://bmjopen.bmj.com/site/about/guidelines.xhtml

Abstract:

Introduction: Preconception health and care strategies can significantly improve maternal and infant outcomes, and thus optimise intergenerational health. The aims of this scoping review are to 1) provide an up-to-date summary of preconception health and care strategies, policies, guidelines, frameworks and recommendations across the UK and Ireland, and 2) explore preconception health and care services, interventions and initiatives in Northern Ireland as a case study.

Methods and analysis: The scoping review of grey literature will be conducted as per the Scoping Review Methods Manual by the Joanna Briggs Institute and the Arksey-O'Malley framework for scoping studies, and reported in line with PRISMA-ScR. Searches will be conducted on Google Advanced Search, OpenAire, NICE, ProQuest and relevant public health websites. Additionally, searches on interventions, services and initiatives provided in Northern Ireland will be supplemented by consultations and audits with key stakeholders to validate findings, identify other potentially eligible resources and ensure breadth of coverage. Data will be extracted into Excel and coded using NVivo, and ≥10% of the data will be double-coded. A narrative approach with content analysis highlighting key themes and concepts will be used to report findings.

Throughout the research cycle, members of the wider public will be involved and engaged with to provide feedback.

Ethics and dissemination: Ethical approval is not required as analyses will be conducted on data available in the public domain. Findings aim to inform future research, practice and decision-making, and aid the development and dissemination of evidence-based actions to optimise preconception health and care potentially leveraging and maximising existing public health programmes.

For peer review only - http://bmjopen.bmj.com/site/about/guidelines.xhtml

Strengths and limitations of this study:

1. This scoping review will be conducted following a systematic process that locates and synthesises evidence on a broad and diverse topic. By transparently mapping the area under investigation, scoping reviews are able to present results in a clear manner, accessible to stakeholders.
2. This scoping review will aim to explore the breadth, rather than depth, of grey literature populating the scene for over a decade (since 2011), allowing the investigation of key themes, concepts and knowledge gaps that can inform future research in the field of preconception health and care. This review will benefit from the contribution and input from those with lived experiences of the preconception period throughout.
3. Additional consultations and audits will be undertaken to validate findings relevant to Northern Ireland, as a case study, to identify other potentially eligible resources and understand any under-researched issues concerning the effectiveness of preconception care services or strategies.
4. Regardless of the measures taken to limit missing relevant citations, it is still possible that potentially eligible resources may have been missed due to the limited number of databases searched, the language restriction applied to the searches and the search terms used. Additionally, relevant resources may have been missed if not available online. As the aim is to review grey literature (i.e., literature not formally published or commercially controlled), any relevant published material will not be included.
5. No quality appraisal will be conducted on the included material, as this is a scoping review aiming to map the breadth of information on the chosen topic.

For peer review only - http://bmjopen.bmj.com/site/about/guidelines.xhtml

Introduction:

Preconception health describes the overall health of non-pregnant individuals of childbearing age, which usually defines individuals aged 18-44 years[1]. The optimisation of preconception health can significantly improve maternal and infant outcomes and, therefore, represents a window of opportunity to improve the health of future generations[2]. Preconception care is the term used to define biomedical, behavioural and social health interventions, services, support and advice provided prior to conception aimed at optimising pregnancy planning and fitness for pregnancy[2,3].

The health status of individuals in the preconception stage can be negatively impacted by risk factors that are often modifiable and/or reversible, and people often have more than one risk factor concurrently[4]. Preconception health risk factors include, for example, parental obesity, long-term physical and mental health conditions, alcohol consumption, smoking, physical inactivity, inadequate dietary habits, low folic acid intake, poor social support, emotional ill-health and low immunisation levels[2,5,6]. While some risk factors are modifiable, such as folic acid intake, others may require further evidence-based support and long-term management to achieve and sustain change, such as weight management in people living with obesity. Risk factors such as socioeconomic status are not easily modifiable and, therefore, demand efforts targeted at vulnerable groups of people affected by inequalities[7]. A comprehensive list of preconception health risk factors is provided in Appendix I, with several of these pertinent risk factors described further below.

Notably, the prevalence of obesity and chronic conditions is increasing. In the UK, approximately 25% of women giving birth live with obesity (body mass index ≥30kg/m^2) at their booking appointment (typically around 12-14 weeks of gestation)[8–12]. Similar proportions are observed in Ireland, where between 19% and 25% of pregnant women live with obesity[13].

Maternal obesity is linked to a greater risk of miscarriage, stillbirth, preterm delivery, macrosomia, gestational diabetes, pre-eclampsia, postpartum haemorrhage, obesity and type II diabetes in the offspring in later life, among other complications[1]. There is also evidence linking paternal obesity with negative pregnancy and child outcomes, reduced

For peer review only - http://bmjopen.bmj.com/site/about/guidelines.xhtml

fertility, and increased birth weight and weight growth velocities[14,15]. Moreover, paternal pre-pregnancy obesity is an influential early-life risk factor for offspring obesity, type II diabetes, insulin resistance and impaired cognitive functions[14,15].

A further risk factor influencing the preconception health of adults of childbearing age is folic acid intake. Folic acid is the synthetic version of folate (vitamin B9), which is critical for embryologic development[16]. It has long been established that folic acid supplementation can reduce the risk of neural tube defects (e.g., spina bifida, anencephaly), preterm delivery and small-for-gestational-age neonates[16,17]. In particular, starting supplementation during pregnancy, as opposed to during the preconception stage, may be too late because the closure of the foetus' neural tube occurs 24-28 days after conception, often before women realise they have conceived[18]. Moreover, although adequate micronutrient intake in the preconception period is recommended, in the UK many women of childbearing age are not nutritionally prepared for pregnancy, even at lower reference nutrient intake levels[19].

Maternal and infant outcomes are also associated with the socioeconomic status of individuals[20]. Compared to women from the least deprived populations, investigations in England have shown that women living in more deprived areas may not only be more likely to be overweight or living with obesity and report the misuse of illicit drugs and smoking, but they may also be less likely to stop smoking during pregnancy, take folic acid supplements and book their first antenatal appointment within 10 weeks of pregnancy as recommended[20].

Preconception care plays a crucial role in the screening, prevention and management of risk factors that may affect maternal and infant outcomes[21]. The provision of preconception care includes the development and implementation of strategies, policies, services, interventions, campaigns, guidelines, frameworks and initiatives. Because preconception care operates at individual, community and population levels, it can positively impact both those who actively plan to conceive and those who do not. This is of particular interest because of the high prevalence of unplanned pregnancies; in the UK, 45% of pregnancies and one-third of births are unplanned or accompanied by feelings of ambivalence[8].

For peer review only - http://bmjopen.bmj.com/site/about/guidelines.xhtml

Recent years have witnessed an increased recognition of the importance of preconception health and care[6]. The investigation of preconception policies, guidelines, recommendations and services provided in six different countries, including the UK, carried out by Shawe and colleagues, concluded that guidelines addressing the preconception phase are heterogeneous[21]. However, as these previous findings are related to searches conducted in 2013, a renewed investigation is now warranted covering the past decade. A further study on preconception guidelines, recommendations and policy reports was conducted in 2021[6]. The review, however, intended to identify preconception indicators, which then formed the basis for the development of a national surveillance system for preconception health in England[22]. A systematic review analysing international clinical guidelines focusing on preconception care was recently conducted to assess the quality of clinical guidelines, the overall recommendations and the evidence recommendations are based on[23]. Findings suggested that guidelines should be improved by broadening the comprehensiveness of the content areas addressed, applying a more rigorous development process and enhancing the acceptability and feasibility of their application[23].

Objectives

From the available literature it is clear that at present it is important to collate the evidence in a coherent and accessible way that enables the identification of common themes, concepts and recommendations, priority areas not yet attended to and strengths and weaknesses of preconception care actions in place across the UK and Ireland. This scoping review will map the evidence in relation to preconception health and care strategies, policies, guidelines, frameworks and recommendations in the UK and Ireland, by summarising recurring themes and concepts underpinning the evidence, identifying gaps in knowledge and exploring future research priorities. This review will also clarify the type of evidence available and the target audience/s of included resources. Northern Ireland will be treated as a case study and, therefore, data on the services, interventions and initiatives provided in the region will be examined, presented and supplemented by consultations and audits with relevant stakeholders.

A systematic scoping approach will be used, as it is appropriate to map the principal themes of a broad research area of interest, describe the breadth of evidence available and

For peer review only - http://bmjopen.bmj.com/site/about/guidelines.xhtml

synthesise heterogeneous evidence[24]. The present protocol will ensure transparency in the research process and overall reliability[25].

Findings from this review will ultimately inform future research, practice and decision-making to optimise preconception health and care, and may help the development of a clear pathway for the promotion of evidence-based advice and support for individuals of childbearing age. Additionally, findings may aid the planning of preconception care actions, potentially leveraging and maximising existing public health programmes.

Research Questions

This scoping review aims to answer the following questions:

a. What strategies, policies, guidelines, frameworks and recommendations have been developed that address preconception health and care for adults in the UK and Ireland between 2011 and 2022?
b. What are the main concepts and themes underpinning strategies, policies, guidelines, frameworks and recommendations that address preconception health and care for adults in the UK and Ireland?
c. How does the evidence from strategies, policies, guidelines, frameworks and recommendations that address preconception care for adults differ across the UK and Ireland?
d. What are, if any, the gaps in the knowledge provided in strategies, policies, services, guidelines, frameworks and recommendations that address preconception health and care for adults in the UK and Ireland, and what areas require further coverage and inquiry?
e. What are, if any, the services, initiatives and interventions provided in Northern Ireland focused on improving preconception health and care in adults?

Answering these questions will provide insights into how the topic of preconception health and care is addressed in the grey literature, summarise the evidence and present an overview of how evidence may differ across the selected countries.

For peer review only - http://bmjopen.bmj.com/site/about/guidelines.xhtml

Methods and analysis:

The proposed scoping review will be conducted in accordance with the Joanna Briggs Institute (JBI) updated methodological guidance for scoping reviews[25], Arksey and O'Malley's framework for conducting scoping studies[24] and reported in line with the Preferred Reporting Items for Systematic Reviews and Meta-analyses extension for scoping reviews (PRISMA-ScR)[26].

A preliminary search of CINAHL, Web of Science, JBI Evidence Synthesis and BMJ Open was conducted and no current or underway scoping reviews on the topic were identified.

Eligibility Criteria

The recent growing interest in preconception health and attention paid to the optimisation of health before pregnancy has led to an increase in preconception care actions by governments and public health organisations, meaning that there is evidence to meet the inclusion criteria for this scoping review.

To develop the eligibility criteria, the Participants-Concept-Context framework was used[27].

Participants: This review will consider resources discussing or reporting on preconception health and care for individuals of childbearing age, and no exclusions in relation to gender, ethnicity, culture, sexuality, health condition/s or disability status will be applied. Therefore, resources addressing individuals with chronic diseases, such as diabetes and epilepsy, will be considered for inclusion. Relevant resources adopting a life-course approach, thus addressing society as a whole, will be considered for inclusion as they also encompass individuals of childbearing age.

Resources may be targeted at the general public, communities, healthcare professionals, provisioners or commissioners, charitable organisations or NGOs, local authorities, policymakers and public health or governmental bodies.

Concept: The present work will summarise evidence underpinning strategies, policies, guidelines, frameworks and recommendations in the UK and Ireland regarding preconception health and care (including topics such as fitness for pregnancy, pregnancy planning and preparation for pregnancy). Furthermore, it will review services, interventions

For peer review only - http://bmjopen.bmj.com/site/about/guidelines.xhtml

and initiatives in Northern Ireland, as an individual case study. A case study approach allows an in-depth analysis of phenomena in a given context[28].

The concept of preconception health will be defined as the overall health of non-pregnant individuals of childbearing age[1], whereas preconception care will be defined as the "services, interventions, support and advice" aimed at optimising pregnancy planning and fitness for pregnancy[2]. Resources explicitly addressing only the interconception period (i.e., the time between conceptions) rather than the preconception period will not be eligible, as this scoping review aims to focus on the preconception period taken in this case to refer to the time before the first pregnancy. The interconception period is of significant importance for maternal and child health, however, a recent policy review covered this specific period[29].

Context: The review will only include resources relevant to the UK and Ireland. Whenever it is unclear whether a resource is relevant, the author/s or relevant organisation/s will be contacted.

Types of Sources

This scoping review will consider grey literature sources not found in the published literature. Grey literature is of interest as it is directed at the public, patients, healthcare professionals and governments, who are the ones who may act directly as a response to recommendations in the documents[6], and is therefore largely accessible also to non-specialist audiences[30]. In this case, the grey literature included refers to strategies, policies, guidelines, frameworks and recommendations addressing preconception health and care in the UK and Ireland. Technical or research reports from government agencies, registered charities or scientific research groups, documents outlining interventions or initiatives from public bodies, articles and guidelines issued by government agencies or professional bodies will be considered for inclusion. In addition, relevant e-learning resources, leaflets and educational booklets will be considered for inclusion.

Journal articles, preprints (journal articles not yet peer-reviewed or published), working papers from research groups, visual or audio content such as television programmes or documentaries (or reviews of this content), academic letters or commentaries, calls for

For peer review only - http://bmjopen.bmj.com/site/about/guidelines.xhtml

participants, presentations and doctoral dissertations will not be considered for inclusion in this scoping review.

Overall, to be considered for inclusion, the material will need to provide concrete, tangible advice and guidance, deliver recommendations or alternatively outline policy actions or strategic plans to improve preconception health and care for adults. Whenever a resource solely mentions preconception health or simply signposts other material, it will not be eligible for inclusion. Although this review aims to summarise grey literature, certain strategies, policies, guidelines, frameworks and recommendations identified during the searches may also be published in the literature.

Details of the inclusion and exclusion criteria applied is presented in Appendix II.

Search Strategy

An initial limited search on Google Advanced Search was undertaken to identify articles on the topic. The text words contained in the titles and abstracts of relevant articles and the index terms used to describe the articles were used to develop a search strategy for database searches. In addition, a subject librarian was consulted to build a transparent and robustly structured search strategy (see Appendix III).

Searches were carried out in May 2022 using the databases Google Advanced Search, National Institute for Health & Clinical Excellence (NICE), OpenAire and ProQuest. Public health and government websites will be also searched (see Appendix IV)[31]. Other grey literature databases such as EThOS, DART, Grey Literature Report, OpenDoar were explored but excluded as not deemed relevant for the study. Boolean operators OR and AND were used. The search strategy, including all identified keywords and index terms, was adapted for each database and/or information source. The reference list of included sources of evidence will be screened for additional sources.

Only documents published in English will be included, as the inquiry is limited to primarily English-speaking countries. Resources published, reviewed or updated during or after 2011 will be considered for inclusion, building upon the timeframe of the previous review across six European countries[21], thus allowing for more than a decade of content to be considered. Material from UK- and/or Ireland-based Royal Colleges and charitable

For peer review only - http://bmjopen.bmj.com/site/about/guidelines.xhtml

organisations and associations with a location or contact address in one of the devolved nations will be deemed eligible for inclusion. Whenever a potentially relevant resource is not readily available or access is restricted, the relevant body or authors will be contacted.

Case study

Additional consultations and audits with relevant stakeholders are favourable to validate findings and ensure breadth of coverage[24]. Therefore, the searches specific to Northern Ireland will be supplemented by audits with stakeholders, including experts in the field of preconception and healthcare professionals (e.g., maternity service providers and clinical leads, consultant midwives, pharmacists). Northern Ireland was deemed of interest as a case study because, although part of the UK, its devolved government has the ability to set its own policies, legislations and agenda.

Overall, these consultations aim to identify other potentially eligible resources, understand any under-researched issues concerning the effectiveness of services or strategies and contextualise any findings. Stakeholders will be sent information about the project prior to a consultation and will be asked to comment on preliminary findings and on whether any relevant resources may be missing. Questions and topic areas discussed during audits will be informed by the findings of the searches conducted. Contributors will also receive communications about the findings of the study, once completed[32].

Selection of Sources of Evidence

Citations identified through searching will be collated and uploaded into Microsoft Excel and duplicates will be removed. Titles and summaries will then be screened by two independent reviewers for assessment against the inclusion criteria for the review. Following the methodology used by Godin and colleagues for examining grey literature[31], only the first 100 results on Google Advanced Search will be reviewed for potentially relevant titles. Potentially relevant sources will be retrieved in full, and the full texts will be assessed in detail against the inclusion criteria by two independent reviewers. At least 10% of resources will be double-coded. Any disagreements that arise between the reviewers at each stage of the selection process will be resolved through discussion, or with an additional reviewer/s, until consensus is reached. Reasons for the exclusion of full-text sources of evidence that do

For peer review only - http://bmjopen.bmj.com/site/about/guidelines.xhtml

not meet the inclusion criteria will be recorded and reported in the scoping review. Overall, the results of the search and the study inclusion process will be reported in full in the final scoping review and presented in a PRISMA-ScR flow diagram[26]. The diagram will illustrate where citations were discarded during the screening process, and a rationale for the exclusion of resources during the full-text screening will also be provided.

Data Extraction

Data will be extracted by the same authors who performed the screening and full-text review to ensure consistency. Data will be extracted from eligible resources independently by two reviewers using a data extraction tool developed by the reviewers on Excel. The data extracted will include specific details about the publication title, publication format (e.g., report, guideline, strategy), target audience (e.g., healthcare professionals, policymakers), overarching aim (e.g., increase awareness among individuals of childbearing age, educate healthcare professionals), participants (e.g., individuals of childbearing age, individuals of childbearing age with diabetes), geographic location, year of publication, review or updates, duration of the strategy, policy or intervention when applicable, and key notes relevant to the research questions. These extracted values were agreed upon after pilot testing the draft extraction form between authors. The data extraction instrument (Appendix V) will be amended if necessary during the data extraction process. The modifications will be detailed in the full scoping review. Any disagreements between the reviewers will be resolved through discussion, or with an additional reviewer/s. When required, an attempt will be made to contact the authors of original resources for clarification if data is unclear, or to request missing or additional data. A follow-up email will be sent after two weeks from the initial contact if no response is received. Thereafter, the missing or unclear data will be documented in the review as such. A critical appraisal of individual sources of evidence will not be undertaken, as this is outside the scope of this review.

Data Analysis and Presentation

The characteristics of included resources will be described in detail (e.g., participants, target audience), a distribution of studies by year will be produced and a map of the available evidence will be presented in tables. Comparisons will be made based on the included

For peer review only - http://bmjopen.bmj.com/site/about/guidelines.xhtml

evidence across the UK and Ireland, and resources addressing preconception health and care services, interventions and initiatives in Northern Ireland will be presented as a case study. Included resources will be coded using NVivo, and at least 10% of the data will be double-coded. A narrative synthesis will highlight the emerging themes and concepts and emphasise how the findings relate to the research questions. The limitations and research gaps will be emphasised and discussed. Authors will adhere to PRISMA-ScR when reporting findings[26].

Patient and Public Involvement and Engagement

The proposed review actively involves a Patient and Public Involvement and Engagement (PPIE) panel of adults aged 18-45 years living in Northern Ireland, named “Healthy Reproductive Years”. The PPIE strategies aim to engage the public as partners and mobilise their existing knowledge and expertise, communicate using a lay language and support a collaborative approach in research by fostering respect and honesty[33]. The PPIE advisory panel was recruited via social media platforms (e.g., Facebook), relevant organisations (e.g., Sure Start), charities and leisure and community centres in Northern Ireland. Recruitment activities aimed to achieve diversity in terms of gender, sexuality, ethnicity, religion, disability, socioeconomic background, education level and health literacy. The PPIE panel was developed to contribute to general discussions and advise on research design, priority setting and dissemination plans which will include the publication of the final report and the co-production of conference materials, a lay summary and an infographic presenting review findings. Representatives have been integrally involved in the development of this protocol and have provided direct feedback on all aspects of it. Collaboration with the PPIE panel will occur via online or in-person meetings and exchange of emails or postal letters.

The reporting of PPIE strategies in this research project will be guided by the Guidance for Reporting Involvement of Patients and the Public (GRIPP) 2 checklist[34]. All PPIE representatives will be reimbursed for their contributions.

For peer review only - http://bmjopen.bmj.com/site/about/guidelines.xhtml

Ethics and dissemination:

Ethical approval is not applicable because this scoping review will analyse publicly accessible resources only.

Findings will be disseminated to service users and relevant stakeholders using diverse approaches, targeted to recipients. Outcomes following this review include a peer-reviewed publication reporting a detailed synthesis of findings and comparisons across countries, a lay summary, a clear and accessible infographic, and presentations directed at conference audiences, stakeholders and the public. The PPIE “Healthy Reproductive Years” panel will be consulted and will inform the dissemination plan. Additionally, a workshop will be held with PPIE representatives to discuss findings.

Discussion and conclusion:

Recent years have seen the rise in the publication of key documents addressing preconception health and care that have the opportunity to positively influence the current landscape by supporting positive health changes, including Making the Case for Preconception Care[2], Missed Periods[35], A Strategy for Maternity Care in Northern Ireland[36] and Women’s Health Strategy for England[37]. These documents emphasise the need to build the evidence base, raise awareness on women’s health and preconception health and care, improve information-sharing and ultimately ensure that all adults of reproductive years are given the tools to optimise their preconception physical, social and psychological health. Recently, it has also been announced that folic acid will be added to non-wholemeal wheat flour in the UK as a way to reduce the incidents of neural tube defects[38]. It is also important to acknowledge that efforts have been made globally. Examples include a policy brief published by the World Health Organization with the aim of improving preconception care[3] and the identification of a condensed set of preconception health indicators in the United States with the aim of better monitoring the health status of women of reproductive age[1].

For peer review only - http://bmjopen.bmj.com/site/about/guidelines.xhtml

Currently, no comprehensive synthesis of the evidence relating to this topic is available in the proposed context of the UK and Ireland. Although this review has potential strengths such as the transparency of the approach adopted, there are also challenges and limitations, such as the lack of a formal critical appraisal of the individual resources, that will be clearly explored and highlighted in the full report.

This scoping review aims to provide an up-to-date systematic mapping of preconception health and care policies, guidelines, strategies, frameworks and recommendations across the UK and Ireland. The proposed review aims to be a scientific basis to aid future activities in the field, impact healthcare practice and policy and make contributions to the design of a clear pathway for the promotion of advice and support for individuals of reproductive age. The proposed scoping review is also expected to contribute to the existing scholarly literature on preconception health and care and inform future research projects. Consequently, findings could positively influence people's behaviours in the preconception phase and accelerate the optimisation of intergenerational health.

For peer review only

For peer review only - http://bmjopen.bmj.com/site/about/guidelines.xhtml

References:

1. Robbins CL, D'Angelo D, Zapata L, et al. Preconception health indicators for public health surveillance. *J Womens Health (Larchmt)*2018;27:430-443. doi:10.1089/jwh.2017.6531
2. Public Health England. Making the case for preconception care. 2018.https://www.gov.uk/government/publications/preconception-care-making-the-case (accessed 22 Apr. 2022).
3. World Health Organization. Policy brief: preconception care – maximising the gains for maternal and child health. 2013.https://www.who.int/publications/i/item/WHO-FWC-MCA-13.02 (accessed 22 Apr. 2022).
4. Evans H, Buck D. Tackling multiple unhealthy risk factors. 2018.https://www.kingsfund.org.uk/sites/default/files/2018-03/Tackling%20multiple%20unhealthy%20risk%20factors%20-%20full%20report.pdf (accessed 22 Apr. 2022).
5. Broussard DL, Sappenfield WB, Fussman C, et al. Core state preconception health indicators: a voluntary, multi-state selection process. *Matern Child Health J*2011;15:158-68. doi:10.1007/s10995-010-0575-x
6. Schoenaker DAJM, Stephenson J, Connolly A, et al. Characterising and monitoring preconception health in England: a review of national population-level indicators and core data sources. *J Dev Orig Health Dis*2021;13:137-150. doi:10.1017/S2040174421000258
7. Public Health England. Maternity high impact area: Reducing the inequality of outcomes for women from Black, Asian and Minority Ethnic (BAME) communities and their babies. 2020.https://assets.publishing.service.gov.uk/government/uploads/system/uploads/attachment_data/file/942480/Maternity_high_impact_area_6_Reducing_the_inequality_of_outcomes_for_women_from_Black__Asian_and_Minority_Ethnic__BAME__communities_and_their_babies.pdf (accessed 26 July 2022).
8. Public Health Agency. Children's health in Northern Ireland. 2019.https://www.publichealth.hscni.net/sites/default/files/2019-

For peer review only - http://bmjopen.bmj.com/site/about/guidelines.xhtml

12/RUAG%20Childrens%20Health%20in%20NI%20-%202018-19%20-%20Dec%202019.pdf (accessed 22 Apr. 2022).

9. Public Health England. Child and maternal health. 2021.https://fingertips.phe.org.uk/profile/child-health-profiles/data#page/1/gid/1938133222 (accessed 3 May 2022).

10. National Services Scotland, National Statistics, Information Services Division. Births in Scottish hospitals. 2019.https://www.isdscotland.org/Health-Topics/Maternity-and-Births/Publications/2019-11-26/2019-11-26-Births-Report.pdf (accessed 3 May 2022).

11. Welsh Government, National Statistics. Maternity and birth statistics: 2020. 2021.https://gov.wales/maternity-and-birth-statistics-2020-html (accessed 3 May 2022).

12. Department of Health. Health survey Northern Ireland: first results 2020/21. 2021.https://www.health-ni.gov.uk/publications/health-survey-northern-ireland-first-results-202021 (accessed 3 May 2022).

13. Institute of Obstetricians and Gynaecologists Royal College of Physicians of Ireland, Clinical Strategy and Programmes Directorate Health Service Executive. Obesity and pregnancy. 2013.https://www.hse.ie/eng/services/publications/clinical-strategy-and-programmes/obesity-and-pregnancy-clinical-practice-guideline.pdf (accessed 21 June 2022).

14. Campbell JM, McPherson NO. Influence of increased paternal BMI on pregnancy and child health outcomes independent of maternal effects: a systematic review and meta-analysis. *Obes Res Clin Pract*2019;13:511-521. doi:10.1016/j.orcp.2019.11.003

15. Lin J, Gu W, Huang H. Effects of paternal obesity on fetal development and pregnancy complications: a prospective clinical cohort study. *Front Endocrinol (Lausanne)*2022;13:826665. doi:10.3389/fendo.2022.826665

16. Valentin M, Coste Mazeau P, Zerah M, et al. Acid folic and pregnancy: A mandatory supplementation. *Ann Endocrinol (Paris)*2018;79:91-94. doi: 10.1016/j.ando.2017.10.001

17. Hodgetts VA, Morris RK, Francis A, et al. Effectiveness of folic acid supplementation in pregnancy on reducing the risk of small-for-gestational age neonates: a population

For peer review only - http://bmjopen.bmj.com/site/about/guidelines.xhtml

study, systematic review and meta-analysis. *BJOG*2015;122:478-90. doi:10.1111/1471-0528.13202

18. Eichholzer M, Tönz O, Zimmermann R. Folic acid: a public-health challenge. *Lancet*2006;367:1352-61. doi:10.1016/S0140-6736(06)68582-6
19. Stephenson J, Heslehurst N, Hall J, et al. Before the beginning: nutrition and lifestyle in the preconception period and its importance for future health. *Lancet*2018;391:1830-1841. doi:10.1016/S0140-6736(18)30311-8
20. Public Health England. Health of women before and during pregnancy: health behaviours, risk factors and inequalities. 2019.https://assets.publishing.service.gov.uk/government/uploads/system/uploads/attachment_data/file/844210/Health_of_women_before_and_during_pregnancy_2019.pdf (accessed 22 Apr. 2022).
21. Shawe J, Delbaere I, Ekstrand M, et al. Preconception care policy, guidelines, recommendations and services across six European countries: Belgium (Flanders), Denmark, Italy, the Netherlands, Sweden and the United Kingdom. *Eur J Contracept Reprod Health Care*2015;20:77-87. doi:10.3109/13625187.2014.990088
22. Schoenaker DAJM, Stephenson J, Smith H, et al. Women's preconception health in England: a report card based on national maternity services data 2018/19. *Lancet* [Preprint]. 2022. http://dx.doi.org/10.2139/ssrn.4163702 (accessed 2 Aug. 2022).
23. Dorney E, Boyle JA, Walker R, et al. A systematic review of clinical guidelines for preconception care. *Semin Reprod Med*2022;40:157-169. doi:10.1055/s-0042-1748190
24. Arksey H, O'Malley L. Scoping studies: towards a methodological framework. *Int J Soc Res Methodol*2005;8:19-32. doi:10.1080/1364557032000119616
25. Peters MDJ, Marnie C, Tricco AC, et al. Updated methodological guidance for the conduct of scoping reviews. *JBI Evid Synth*2020;18:2119-2126. doi: 10.11124/JBIES-20-00167
26. Tricco AC, Lillie E, Zarin W, et al. PRISMA extension for scoping reviews (PRISMA-ScR): Checklist and Explanation. *Ann Intern Med*2018;169:467-473. doi:10.7326/M18-0850

For peer review only - http://bmjopen.bmj.com/site/about/guidelines.xhtml

27. Peters MDJ, Godfrey C, McInerney P, et al. *Chapter 11: Scoping Reviews*. In: Aromataris E, Munn Z (Editors). JBI Manual for evidence synthesis. Adelaide: The Joanna Briggs Institute 2020
28. Gagnon, YC. *The case study as a research method*. Canada: Presses de l'Universite du Quebec 2010.
29. Watson D, Jacob CM, Giles G, et al. A scoping review of nutritional interventions and policy guidelines in the interconception period for prevention of noncommunicable diseases. *Reprod Female Child Health*2022. doi:10.1002/rfc2.8
30. Pappas C, Williams I. Grey literature: its emerging importance. *J Hosp Librariansh*2011,11:228-234. Doi:10.1080/15323269.2011.587100
31. Godin K, Stapleton J, Kirkpatrick SI, et al. Applying systematic review search methods to the grey literature: a case study examining guidelines for school-based breakfast programs in Canada. *Syst Rev*2015;4:138. doi:10.1186/s13643-015-0125-0
32. Joosten YA, Israel TL, Williams NA, et al. Community engagement studios: a structured approach to obtaining meaningful input from stakeholders to inform research. *Acad Med*2015;90:1646-50. doi:10.1097/ACM.0000000000000794
33. Canadian Institutes of Health Research. Strategy for Patient-Oriented Research. Capacity development framework. 2015.https://cdn.dal.ca/content/dam/dalhousie/pdf/research-services/REB/Newsletter/spor_capacity_development_framework-en.pdf (accessed 26 May 2022).
34. Staniszewska S, Brett J, Simera I, et al. GRIPP2 reporting checklists: tools to improve reporting of patient and public involvement in research. *Res Involv Engagem*2017;3:13. doi:10.1136/bmj.j3453
35. Sher J. Missed periods. 2016.https://www.nhsggc.org.uk/media/237840/missed-periods-j-sher-may-2016.pdf (accessed 5 May 2022).
36. Department of Health, Social Services and Public Safety. A strategy for maternity care in Northern Ireland. 2012.https://www.health-ni.gov.uk/sites/default/files/publications/dhssps/maternitystrategy.pdf (accessed 5 May 2022).

For peer review only - http://bmjopen.bmj.com/site/about/guidelines.xhtml

37. Department of Health & Social Care. Women's Health Strategy for England. 2022.https://www.gov.uk/government/publications/womens-health-strategy-for-england/womens-health-strategy-for-england (accessed 26 July 2022).

38. UK Government. Proposal to add folic acid to flour: consultation response. 2021.https://www.gov.uk/government/consultations/adding-folic-acid-to-flour/outcome/proposal-to-add-folic-acid-to-flour-consultation-response (accessed 20 July 2022).

For peer review only

For peer review only - http://bmjopen.bmj.com/site/about/guidelines.xhtml

Authors' Contributions:

EC, LM, MM, LK discussed the protocol and finalised the methodological process of the scoping review. EC drafted the protocol, which was finalised with input from LM, MM, LK, KAE and DS. DT and TS, members of the Patient and Public Involvement and Engagement "Healthy Reproductive Years" panel, also helped shape the research and reviewed the protocol. All authors have seen and approved the final version of the protocol for publication.

Acknowledgements:

The authors would like to acknowledge the contribution of the Patient and Public Involvement and Engagement "Healthy Reproductive Years" panel for their assistance.

Funding Statement:

This work is supported by Queen's University Belfast as part of a PhD studentship for EC. EC is funded by the Department for the Economy NI. DS is supported by the National Institute for Health and Social Care Research (NIHR) Southampton Biomedical Research Centre [IS-BRC-1215-20004]). The views expressed are those of the author(s) and not necessarily those of the NIHR or the Department of Health and Social Care.

Competing Interests Statement:

There is no conflict of interest in this project.

Keywords:

Pre-conception; reproductive health; pregnancy; grey literature.

For peer review only

Appendices:

Appendix I: Preconception risk factors

**Social**		
Adverse childhood experiences<br>Belonging to an ethnic minority<br>Deprivation<br>Domestic abuse (physical and emotional)	Environmental tobacco smoke and second-hand smoke exposure<br>Exposure to toxic or hazardous substances<br>Financial insecurity or unemployment<br>Inadequate support from family or social network	Low educational attainment<br>Migration status<br>Unsuitable access to quality healthcare and dentalcare<br>Unsuitable housing
**Behavioural**		
Alcohol misuse<br>Eating disorders<br>Inadequate dietary habits	Lack of family planning/pregnancy intention<br>Physical inactivity or sedentary behaviours	Smoking<br>Substance misuse<br>Use of teratogenic medications
**Biomedical**		
Fertility issues<br>Folic acid or other vitamin deficiency<br>General health status<br>Genetic risks<br>Infections (e.g., sexually transmitted diseases)	Low immunisation levels<br>Mental health conditions (e.g., anxiety, depression)<br>Parental age<br>Parental obesity or overweight, parental underweight	Physical health conditions (e.g., diabetes mellitus, epilepsy, endometriosis, chronic hypertension)<br>Previous pregnancy and obstetric complications (e.g., previous foetal death, miscarriage, stillbirth, preterm birth)

References for the table: [4–6]

For peer review only - http://bmjopen.bmj.com/site/about/guidelines.xhtml

Appendix II: Inclusion and exclusion criteria for a scoping review investigating strategies, policies, services, guidelines, frameworks and recommendations that address preconception health and care for adults in the UK and Ireland.

Inclusion	Exclusion
The resource is one of the following: strategies, policies, guidelines, frameworks and recommendations, including technical or research reports from government agencies, registered charities or scientific research groups, documents outlining interventions or initiatives from public bodies, articles and guidelines issued by government agencies, Royal Colleges and professional bodies, e-learning resources, leaflets and educational booklets. If the resource is a service, intervention or initiative it will be included only if pertinent to Northern Ireland.	The resource is one of the following: journal articles, preprints (journal articles not yet peer-reviewed or published), working papers from research groups, visual or audio content (or reviews of visual or audio content), academic letters or commentaries, calls for participants, presentations and doctoral dissertations. If the resource is a service, intervention or initiative it will be excluded if not pertinent to Northern Ireland.
The resource addresses preconception health and care.	The resource does not address preconception health and care. This includes if the resource explicitly addresses sexual health, interconception health and care or pregnancy.
The resource is relevant to the UK or Ireland.	The resource is not relevant to the UK or Ireland.
The resource was published, reviewed or updated during or after January 2011.	The resource was published, reviewed or updated before January 2011.
The resource was published in English.	The resource was published in any language other than English.
The resource presents unique insights and does not simply report, cite or signpost other included resources.	The resource mentions preconception health and care only by reporting, citing or signposting included resources, and therefore duplicates findings of an earlier version of the resource.
The resource is currently available.	The resource is not currently available (e.g., guideline being updated).

For peer review only - http://bmjopen.bmj.com/site/about/guidelines.xhtml

Appendix III: Search strategy

List of searches conducted on Advanced Google Search for Northern Ireland.

	**Search terms**
1	~preconception AND "Northern Ireland" OR NI
2	~preconception AND "Northern Ireland" AND policy OR service OR intervention OR guideline OR strategy OR initiative
3	"preconception health" OR "preconception care" AND "Northern Ireland" OR NI
4	~pre-pregnancy AND "Northern Ireland" OR NI AND policy OR service OR intervention OR guideline OR strategy OR initiative
5	~preconception AND "Northern Ireland" OR NI AND ~charity
6	~preconception AND "Health and Social Care Trust" OR HSCT
7	"pregnancy planning" OR "planning a baby" OR "preparation for pregnancy" OR "preparation for parenthood" OR "pregnancy intention" OR "expecting mothers" OR "expectant mothers" OR "conceive" OR "future pregnancy" AND "Northern Ireland"
8	"reproductive health" OR "sexual health" AND "Northern Ireland" AND policy OR service OR intervention OR guideline OR strategy OR initiative

Results limited based on language (English) and country (United Kingdom).

For peer review only

For peer review only - http://bmjopen.bmj.com/site/about/guidelines.xhtml

Appendix IV: Additional intended sources and websites

Source	Website	Context
National Institute for Health Research	https://www.nihr.ac.uk/	UK
British National Health Service (NHS)	https://www.nhs.uk/	UK
Royal College of Obstetricians and Gynaecologists	https://www.rcog.org.uk/	UK
Tommy's	https://www.tommys.org/	England, Scotland, Wales
Office for Health Improvement & Disparities	https://www.gov.uk/government/organisations/office-for-health-improvement-and-disparities	England
Public Health Wales	https://phw.nhs.wales/	Wales
Public Health Scotland	https://www.publichealthscotland.scot/	Scotland
NHS Inform	https://www.nhsinform.scot/	Scotland
Health Well	https://healthwell.eani.org.uk	Northern Ireland
Department of Health (Northern Ireland)	https://www.health-ni.gov.uk/	Northern Ireland
Sexual Health NI	https://www.sexualhealthni.info/	Northern Ireland
NI DIRECT	https://www.nidirect.gov.uk/	Northern Ireland
Public Health Agency	https://www.publichealth.hscni.net/	Northern Ireland
Health Service Executive IE	https://www.hse.ie/eng/	Ireland
Health Information and Quality Authority	https://www.hiqa.ie/areas-we-work/health-information/data-collections/all-ireland-public-health-repository	Ireland

For peer review only - http://bmjopen.bmj.com/site/about/guidelines.xhtml

Appendix V: Data extraction instrument

**Data to be extracted**	**Notes to reviewer**
Title	
Year of publication	
Resource format	e.g., report, intervention, strategy, policy
Source	e.g., Google Advanced Search, NICE, OpenAire
Year of publication/Year of most recent update.	
Duration	When applicable, the duration of the strategy, policy or intervention will be reported
Participants	Target population
Context	Country
Target audience	e.g., healthcare professionals, policymakers
Overarching aim	e.g., educate, increase awareness
Key notes on topics/content	

When extracting data, the initials of the reviewers and the date when the data extraction occurred will also be reported.

For peer review only

Preferred Reporting Items for Systematic reviews and Meta-Analyses extension for Scoping Reviews (PRISMA-ScR) Checklist

SECTION	ITEM	PRISMA-ScR CHECKLIST ITEM	REPORTED ON PAGE #
**TITLE**			
Title	1	Identify the report as a scoping review.	i
**ABSTRACT**			
Structured summary	2	Provide a structured summary that includes (as applicable): background, objectives, eligibility criteria, sources of evidence, charting methods, results, and conclusions that relate to the review questions and objectives.	ii
**INTRODUCTION**			
Rationale	3	Describe the rationale for the review in the context of what is already known. Explain why the review questions/objectives lend themselves to a scoping review approach.	1-3
Objectives	4	Provide an explicit statement of the questions and objectives being addressed with reference to their key elements (e.g., population or participants, concepts, and context) or other relevant key elements used to conceptualize the review questions and/or objectives.	3-4
**METHODS**			
Protocol and registration	5	Indicate whether a review protocol exists; state if and where it can be accessed (e.g., a Web address); and if available, provide registration information, including the registration number.	NA
Eligibility criteria	6	Specify characteristics of the sources of evidence used as eligibility criteria (e.g., years considered, language, and publication status), and provide a rationale.	5-6
Information sources*	7	Describe all information sources in the search (e.g., databases with dates of coverage and contact with authors to identify additional sources), as well as the date the most recent search was executed.	7
Search	8	Present the full electronic search strategy for at least 1 database, including any limits used, such that it could be repeated.	21
Selection of sources of evidence†	9	State the process for selecting sources of evidence (i.e., screening and eligibility) included in the scoping review.	8-9
Data charting process‡	10	Describe the methods of charting data from the included sources of evidence (e.g., calibrated forms or forms that have been tested by the team before their use, and whether data charting was done independently or in duplicate) and any processes for obtaining and confirming data from investigators.	9-10
Data items	11	List and define all variables for which data were sought and any assumptions and simplifications made.	9
Critical appraisal of individual sources of evidence§	12	If done, provide a rationale for conducting a critical appraisal of included sources of evidence; describe the methods used and how this information was used in any data synthesis (if appropriate).	NA
Synthesis of results	13	Describe the methods of handling and summarizing the data that were charted.	9-10

St. Michael's
Inspired Care. Inspiring Science.

For peer review only - http://bmjopen.bmj.com/site/about/guidelines.xhtml

SECTION	ITEM	PRISMA-ScR CHECKLIST ITEM	REPORTED ON PAGE #
**RESULTS**			
Selection of sources of evidence	14	Give numbers of sources of evidence screened, assessed for eligibility, and included in the review, with reasons for exclusions at each stage, ideally using a flow diagram.	NA
Characteristics of sources of evidence	15	For each source of evidence, present characteristics for which data were charted and provide the citations.	NA
Critical appraisal within sources of evidence	16	If done, present data on critical appraisal of included sources of evidence (see item 12).	NA
Results of individual sources of evidence	17	For each included source of evidence, present the relevant data that were charted that relate to the review questions and objectives.	NA
Synthesis of results	18	Summarize and/or present the charting results as they relate to the review questions and objectives.	NA
**DISCUSSION**			
Summary of evidence	19	Summarize the main results (including an overview of concepts, themes, and types of evidence available), link to the review questions and objectives, and consider the relevance to key groups.	NA
Limitations	20	Discuss the limitations of the scoping review process.	NA
Conclusions	21	Provide a general interpretation of the results with respect to the review questions and objectives, as well as potential implications and/or next steps.	11-12
**FUNDING**			
Funding	22	Describe sources of funding for the included sources of evidence, as well as sources of funding for the scoping review. Describe the role of the funders of the scoping review.	18

JBI = Joanna Briggs Institute; PRISMA-ScR = Preferred Reporting Items for Systematic reviews and Meta-Analyses extension for Scoping Reviews.
* Where *sources of evidence* (see second footnote) are compiled from, such as bibliographic databases, social media platforms, and Web sites.
† A more inclusive/heterogeneous term used to account for the different types of evidence or data sources (e.g., quantitative and/or qualitative research, expert opinion, and policy documents) that may be eligible in a scoping review as opposed to only studies. This is not to be confused with *information sources* (see first footnote).
‡ The frameworks by Arksey and O'Malley (6) and Levac and colleagues (7) and the JBI guidance (4, 5) refer to the process of data extraction in a scoping review as data charting.
§ The process of systematically examining research evidence to assess its validity, results, and relevance before using it to inform a decision. This term is used for items 12 and 19 instead of "risk of bias" (which is more applicable to systematic reviews of interventions) to include and acknowledge the various sources of evidence that may be used in a scoping review (e.g., quantitative and/or qualitative research, expert opinion, and policy document).

From: Tricco AC, Lillie E, Zarin W, O'Brien KK, Colquhoun H, Levac D, et al. PRISMA Extension for Scoping Reviews (PRISMAScR): Checklist and Explanation. Ann Intern Med. 2018;169:467–473. doi: 10.7326/M18-0850.

For peer review only - http://bmjopen.bmj.com/site/about/guidelines.xhtml

BMJ Open

Preconception health and care policies, strategies and guidelines in the UK and Ireland: A scoping review protocol

Journal:	*BMJ Open*
Manuscript ID	bmjopen-2022-067822.R1
Article Type:	Protocol
Date Submitted by the Author:	09-Mar-2023
Complete List of Authors:	Cassinelli, Emma ; Queen's University Belfast,<br>McKinley, Michelle C.; Queen's University Belfast<br>Kent, Lisa; Queen's University Belfast, Centre for Public Health<br>Eastwood, Kelly-Ann; University Hospitals Bristol and Weston NHS Foundation Trust; Queen's University Belfast<br>Schoenaker , Danielle; University of Southampton,<br>Trew, David; Patient and Public Involvement and Engagement<br>Stoikidou, Theano ; Patient and Public Involvement and Engagement<br>McGowan, Laura; Queen's University Belfast
**Primary Subject Heading**:	Public health
Secondary Subject Heading:	Health policy, Sexual health
Keywords:	PUBLIC HEALTH, Maternal medicine < OBSTETRICS, Reproductive medicine < GYNAECOLOGY, Protocols & guidelines < HEALTH SERVICES ADMINISTRATION & MANAGEMENT

SCHOLARONE™
Manuscripts

Title: Preconception health and care policies, strategies and guidelines in the UK and Ireland: A scoping review protocol

Authors: Emma Hope Cassinelli[1], Prof Michelle McKinley[1], Dr Lisa Kent[1], Dr Kelly-Ann Eastwood [1,2], Dr Danielle Schoenaker[3], David Trew[4], Theano Stoikidou[4], Dr Laura McGowan[1]

1. Centre for Public Health (Institute for Global Food Security), School of Medicine, Dentistry and Biomedical Sciences, Queen's University Belfast, Belfast (United Kingdom).

2. University Hospitals Bristol NHS Foundation Trust, Bristol (United Kingdom).

3. NIHR Southampton Biomedical Research Centre, University of Southampton and University Hospital Southampton NHS Foundation Trust, Southampton (United Kingdom).

4 Patient and Public Involvement and Engagement "Healthy Reproductive Years" Panel

Corresponding author: Dr Laura McGowan, Lecturer in Nutrition and Behaviour Change.

Postal address: Institute for Global Food Security (Centre for Public Health), Queen's University Belfast, Institute of Clinical Sciences, Block A. Grosvenor Road, Belfast, BT12 6BJ, United Kingdom.

Email address: laura.mcgowan@qub.ac.uk

For peer review only

Abstract:

Introduction: Preconception health and care strategies can significantly improve maternal and infant outcomes, and thus optimise intergenerational health. The aims of this scoping review are to 1) provide an up-to-date summary of preconception health and care strategies, policies, guidelines, frameworks and recommendations across the UK and Ireland, and 2) explore preconception health and care services, interventions and initiatives in Northern Ireland as a case study.

Methods and analysis: The scoping review of grey literature will be conducted as per the Scoping Review Methods Manual by the Joanna Briggs Institute and the Arksey-O'Malley framework for scoping studies, and reported in line with PRISMA-ScR. Searches were conducted on Google Advanced Search, OpenAire, NICE, ProQuest and relevant public health websites in May 2022. Additionally, searches on interventions, services and initiatives provided in Northern Ireland will be supplemented by consultations and audits with key stakeholders to validate findings, identify other potentially eligible resources and ensure breadth of coverage. Data will be extracted into Excel and coded using NVivo, and ≥10% of the data will be double-coded. A narrative approach with content analysis highlighting key themes and concepts will be used to report findings.

Throughout the research cycle, members of the wider public will be involved and engaged with to provide feedback.

Ethics and dissemination: Ethical approval is not required as analyses will be conducted on data available in the public domain. Findings will be shared with relevant stakeholders with the aim to inform future research, practice and decision-making, and disseminated through a peer-reviewed publication, conference presentations and infographics. Dissemination plans will be informed by the "Healthy Reproductive years" PPIE advisory panel.

For peer review only - http://bmjopen.bmj.com/site/about/guidelines.xhtml

Strengths and limitations of this study:

1. This scoping review will be conducted following a systematic process to transparently locate and synthesise evidence, informed by the Joanna Briggs Institute updated methodological guidance and Arksey and O'Malley's framework, and will be reported in line with the Preferred Reporting Items for Systematic Reviews and Meta-analyses extension for scoping reviews (PRISMA-ScR).
2. This review will incorporate contributions and feedback from those with lived experiences of the preconception period throughout.
3. Consultations and audits will be undertaken to validate findings relevant to Northern Ireland, as a case study, identify other potentially eligible resources and understand any under-researched issues concerning the effectiveness of preconception care services or strategies.
4. No quality appraisal will be conducted on the included material, as this is a scoping review aiming to map the breadth of information on the chosen topic.

For peer review only

Introduction:

Preconception health describes the overall health of non-pregnant individuals of childbearing age, which usually defines individuals aged 18-44 years[1]. The optimisation of preconception health can significantly improve maternal and infant outcomes and, therefore, represents a window of opportunity to improve the health of future generations[2]. Preconception care is the term used to define biomedical, behavioural and social health interventions, services, support and advice provided prior to conception aimed at optimising pregnancy planning and fitness for pregnancy[2,3].

The health status of individuals in the preconception stage can be negatively impacted by risk factors that are often modifiable and/or reversible, and people often have more than one risk factor concurrently[4]. Preconception health risk factors include, for example, parental obesity, long-term physical and mental health conditions, alcohol consumption, smoking, physical inactivity, inadequate dietary habits, low folic acid intake, poor social support, emotional ill-health and low immunisation levels[2,5,6]. While some risk factors are modifiable, such as folic acid intake, others may require further evidence-based support and long-term management to achieve and sustain change, such as weight management in people living with obesity. Risk factors such as socioeconomic status are not easily modifiable and, therefore, demand efforts targeted at vulnerable groups of people affected by inequalities[7]. For example, compared to women from the least deprived populations, investigations in England have shown that women living in more deprived areas may not only be more likely to be overweight or living with obesity and report the misuse of illicit drugs and smoking, but they may also be less likely to stop smoking during pregnancy, take folic acid supplements and book their first antenatal appointment within 10 weeks of pregnancy as recommended[8]. A comprehensive list of preconception health risk factors is provided in Appendix I.

Preconception care plays a crucial role in the screening, prevention and management of risk factors that may affect maternal and infant outcomes[9]. The provision of preconception care includes the development and implementation of strategies, policies, services, interventions, campaigns, guidelines, frameworks and initiatives. Because preconception

For peer review only - http://bmjopen.bmj.com/site/about/guidelines.xhtml

care operates at individual, community and population levels, it can positively impact both those who actively plan to conceive and those who do not. This is of particular interest because of the high prevalence of unplanned pregnancies; in the UK, 45% of pregnancies and one-third of births are unplanned or accompanied by feelings of ambivalence[2, 10].

Recent years have witnessed an increased recognition of the importance of preconception health and care[6], in both the academic field and policy (e.g., Making the Case for Preconception Care[2]), suggesting that recent publicly accessible resources may have been produced but not yet reviewed in this capacity.

Shawe and colleagues conducted an investigation of preconception policies, guidelines, recommendations and services available in six different countries, including the UK, to provide a baseline comparison of current materials [9]. They found variations between recommendations (e.g., folic acid supplementation) and concluded that guidelines addressing the preconception phase are overall heterogeneous[9]. As these previous findings are related to searches conducted in 2013, a renewed investigation is now warranted covering the past decade.

A further study on preconception guidelines, recommendations and policy reports was conducted in 2021[6]. The review, however, intended to inform the reporting of population-level preconception health in England and identify preconception indicators, which then formed the basis for the development of a national surveillance system for preconception health in England[11]. Sixty-six indicators were identified across 12 domains, namely wider determinants of health, health care, environmental exposures, reproductive health and family planning, health behaviours, cervical screening, immunisation and infections, mental and physical health, medications and genetic risk[6].

A systematic review was recently conducted to analyse freely accessible international clinical guidelines focusing on preconception care[12]. They identified eleven guidelines and assessed the quality of the guidelines themselves using the AGREE II tool, the overall recommendations and the evidence recommendations are based on[12]. Findings suggested that high-quality guidelines are lacking and that guidelines should be improved by broadening the comprehensiveness of the content areas addressed, applying a more rigorous development process and enhancing the acceptability and feasibility of their

For peer review only - http://bmjopen.bmj.com/site/about/guidelines.xhtml

application[12]. No previous review was found that included both proposed locations of this scoping review: UK and Ireland.

Objectives

From the available literature it is clear that at present it is important to collate the evidence in a coherent and accessible way that enables the identification of common themes, concepts and recommendations, priority areas not yet attended to and strengths and weaknesses of preconception care actions in place across the UK and Ireland. This scoping review aims to build upon existing research and map the evidence in relation to preconception health and care strategies, policies, guidelines, frameworks and recommendations in the UK and Ireland, by summarising recurring themes and concepts underpinning the evidence, identifying gaps in knowledge and exploring future research priorities. This review will also clarify the type of evidence available and the target audience/s of included resources. Northern Ireland will be treated as a case study and, therefore, data on the services, interventions and initiatives provided in the region will be examined, presented and supplemented by consultations and audits with relevant stakeholders.

A systematic scoping approach will be used, as it is appropriate to map the principal themes of a broad research area of interest, describe the breadth of evidence available and synthesise heterogeneous evidence[13]. The present protocol will ensure transparency in the research process and overall reliability[14].

Findings from this review will ultimately inform future research, practice and decision-making to optimise preconception health and care, and may help the development of a clear pathway for the promotion of evidence-based advice and support for individuals of childbearing age. Additionally, findings may aid the planning of preconception care actions, potentially leveraging and maximising existing public health programmes.

Research Questions

This scoping review aims to answer the following questions:

For peer review only

a. What strategies, policies, guidelines, frameworks and recommendations have been developed that address preconception health and care for adults in the UK and Ireland between 2011 and 2022?
b. What are the main concepts and themes underpinning strategies, policies, guidelines, frameworks and recommendations that address preconception health and care for adults in the UK and Ireland?
c. How does the evidence from strategies, policies, guidelines, frameworks and recommendations that address preconception care for adults differ across the UK and Ireland?
d. What are, if any, the gaps in the knowledge provided in strategies, policies, services, guidelines, frameworks and recommendations that address preconception health and care for adults in the UK and Ireland, and what areas require further coverage and inquiry?
e. What are, if any, the services, initiatives and interventions provided in Northern Ireland focused on improving preconception health and care in adults?

Answering these questions will provide insights into how the topic of preconception health and care is addressed in the grey literature, summarise the evidence and present an overview of how evidence may differ across the selected countries.

Methods and analysis:

The proposed scoping review will be conducted in accordance with the Joanna Briggs Institute (JBI) updated methodological guidance for scoping reviews[14], Arksey and O'Malley's framework for conducting scoping studies[13] and reported in line with the Preferred Reporting Items for Systematic Reviews and Meta-analyses extension for scoping reviews (PRISMA-ScR)[15].

A preliminary search of CINAHL, Web of Science, JBI Evidence Synthesis and BMJ Open was conducted and no current or underway scoping reviews on the topic were identified.

For peer review only

For peer review only - http://bmjopen.bmj.com/site/about/guidelines.xhtml

Eligibility Criteria

The recent growing interest in preconception health and attention paid to the optimisation of health before pregnancy has led to an increase in preconception care actions by governments and public health organisations (e.g., folic acid fortification), meaning that there is evidence to meet the inclusion criteria for this scoping review.

To develop the eligibility criteria, the Participants-Concept-Context framework outlined by Peters and colleagues was used[16], as described below. These criteria were developed in accordance with the study objectives and informed by the meaningful findings highlighted by Schoenaker and colleagues[6] and the critical discourse presented in resources such as Making the Case for Preconception Care [2].

Participants: This review will consider resources discussing or reporting on preconception health and care for individuals of childbearing age, and no exclusions in relation to gender, ethnicity, culture, sexuality, health condition/s or disability status will be applied. Therefore, resources addressing individuals with chronic diseases, such as diabetes and epilepsy, will be considered for inclusion. Relevant resources adopting a life-course approach, thus addressing society as a whole, will be considered for inclusion as they also encompass individuals of childbearing age.

Concept: The present work will summarise evidence underpinning strategies, policies, guidelines, frameworks and recommendations in the UK and Ireland regarding preconception health and care (see Appendix II Glossary of terms), including topics such as fitness for pregnancy, pregnancy planning and preparation for pregnancy. Furthermore, it will review services, interventions and initiatives in Northern Ireland, as an individual case study. A case study approach allows an in-depth analysis of phenomena in a given context[17].

Resources explicitly addressing onlythe interconception period (see Appendix II Glossary of terms) will not be considered eligible, also to avoid duplication of research due to a recent policy review covering this specific period[18]. *Context*: The review will only include resources relevant to the UK and Ireland.

For peer review only - http://bmjopen.bmj.com/site/about/guidelines.xhtml

Whenever it is unclear whether a resource is relevant, the author/s or relevant organisation/s will be contacted.

Types of Sources

This scoping review will consider grey literature sources not found in the published literature. In this case, the grey literature included refers to strategies, policies, guidelines, frameworks and recommendations addressing preconception health and care in the UK and Ireland. Technical or research reports from government agencies, registered charities or scientific research groups, documents outlining interventions or initiatives from public bodies, articles and guidelines issued by government agencies or professional bodies will be considered for inclusion. In addition, leaflets and educational booklets will be considered for inclusion, as well as relevant e-learning resources due to their increasing popularity among healthcare professionals and ability to provide information that is easily understandable. Journal articles, preprints (journal articles not yet peer-reviewed or published), working papers from research groups, visual or audio content such as television programmes or documentaries (or reviews of this content), academic letters or commentaries, calls for participants, presentations and doctoral dissertations will not be considered for inclusion in this scoping review.

Grey literature, including but limited to policies, was deemed of interest as opposed to published literature as it is directed at the public, patients, healthcare professionals and governments, who are the ones who may act directly as a response to recommendations in the documents to review[6]. Grey literature holds the potential to disseminate findings to wider audiences as it is largely accessible also to non-specialist audiences[19,20], and can provide a current, balanced and comprehensive view of the evidence[21].

Overall, to be considered for inclusion, the material will need to provide concrete, tangible advice and guidance, deliver recommendations or alternatively outline policy actions or strategic plans to improve preconception health and care for adults of childbearing age. Whenever a resource solely mentions preconception health or simply signposts other material, it will not be eligible for inclusion. Although this review aims to summarise grey

For peer review only - http://bmjopen.bmj.com/site/about/guidelines.xhtml

literature, certain strategies, policies, guidelines, frameworks and recommendations identified during the searches may also be published in the literature.

Details of the inclusion and exclusion criteria applied is presented in Appendix III.

Search Strategy

An initial limited search on Google Advanced Search was undertaken to identify articles on the topic. The text words contained in the titles and abstracts of relevant articles and the index terms used to describe the articles were used to develop a search strategy for database searches. In addition, a subject librarian was consulted to build a transparent and robustly structured search strategy (see Appendix IV).

Searches were carried out in May 2022 using the databases Google Advanced Search, National Institute for Health & Clinical Excellence (NICE), OpenAire and ProQuest. Public health and government websites were also searched (see Appendix V). This process of locating sources was informed by Godin *et al*.[22]. Other grey literature databases such as EThOS, DART, Grey Literature Report, OpenDoar were explored but excluded as not deemed relevant for the study. Boolean operators OR and AND were used. The search strategy, including all identified keywords and index terms, was adapted for each database and/or information source. The reference list of included sources of evidence will be screened for additional sources. Consultations with experts will be conducted in March and April 2023 to inform research and contextualise findings. Therefore, they will be conducted once preliminary findings from the scoping exercise are collated.

Only documents published in English will be included, as the inquiry is limited to primarily English-speaking countries. Resources published, reviewed or updated during or after 2011 will be considered for inclusion, building upon the timeframe of the previous review across six European countries[9], thus allowing for more than a decade of content to be considered. Material from UK- and/or Ireland-based Royal Colleges and charitable organisations and associations with a location or contact address in one of the devolved nations will be deemed eligible for inclusion. Whenever a potentially relevant resource is not readily available or access is restricted, the relevant body or authors will be contacted.

For peer review only - http://bmjopen.bmj.com/site/about/guidelines.xhtml

Case study

Consultations and audits with relevant stakeholders are favourable to validate findings and ensure breadth of coverage[13]. Therefore, the searches specific to Northern Ireland will be supplemented by audits with stakeholders, including experts in the field of preconception and healthcare professionals (e.g., maternity service providers and clinical leads, consultant midwives, pharmacists). Northern Ireland was deemed of interest as a case study because, although part of the UK, its devolved government has the ability to set its own policies, legislations and agenda. Moreover, preconception health is increasingly receiving importance in Northern Ireland, which has led to the development of a Strategy for maternity care[23], increased funding for the development of perinatal mental health teams across Health and Social Care Trusts[24] and support for the introduction of the mandatory fortification of flour with folic acid, for example[25].

Overall, these consultations aim to identify other potentially eligible resources, understand any under-researched issues concerning the effectiveness of services or strategies and contextualise any findings. Stakeholders will be sent information about the project prior to a consultation and will be asked to comment on preliminary findings and on whether any relevant resources may be missing. Questions and topic areas discussed during audits will be informed by the findings of the searches conducted. Contributors will also receive communications about the findings of the study, once completed[26].

Selection of Sources of Evidence

Citations identified through searching will be collated and uploaded into Microsoft Excel and duplicates will be removed. Titles and summaries will then be screened by two independent reviewers for assessment against the inclusion criteria for the review. Following the methodology used by Godin and colleagues for examining grey literature[22], only the first 100 results on Google Advanced Search will be reviewed for potentially relevant titles. Potentially relevant sources will be retrieved in full, and the full texts will be assessed in detail against the inclusion criteria by two independent reviewers. At least 10% of resources will be double-coded. Any disagreements that arise between the reviewers at each stage of the selection process will be resolved through discussion, or with an additional reviewer/s,

For peer review only - http://bmjopen.bmj.com/site/about/guidelines.xhtml

until consensus is reached. Reasons for the exclusion of full-text sources of evidence that do not meet the inclusion criteria will be recorded and reported in the scoping review. Overall, the results of the search and the study inclusion process will be reported in full in the final scoping review and presented in a PRISMA-ScR flow diagram[15]. The diagram will illustrate where citations were discarded during the screening process, and a rationale for the exclusion of resources during the full-text screening will also be provided.

Data Extraction

Data will be extracted by the same authors who performed the screening and full-text review to ensure consistency. Data will be extracted from eligible resources independently by two reviewers using a data extraction tool developed by the reviewers on Excel. The data extracted will include specific details about the publication title, publication format (e.g., report, guideline, strategy), target audience (e.g., healthcare professionals, policymakers), overarching aim (e.g., increase awareness among individuals of childbearing age, educate healthcare professionals), participants (e.g., individuals of childbearing age, individuals of childbearing age with diabetes), geographic location, year of publication, review or updates, duration of the strategy, policy or intervention when applicable, and key notes relevant to the research questions. These extracted values were agreed upon after pilot testing the draft extraction form between authors (initial example shown in Appendix VI). The revised data extraction instrument (Appendix VII) will be amended if necessary during the data extraction process. The modifications will be detailed in the full scoping review. Any disagreements between the reviewers will be resolved through discussion, or with an additional reviewer/s. When required, an attempt will be made to contact the authors of original resources for clarification if data is unclear, or to request missing or additional data. A follow-up email will be sent after two weeks from the initial contact if no response is received. Thereafter, the missing or unclear data will be documented in the review as such. A critical appraisal of individual sources of evidence will not be undertaken, as this is not a requirement for scoping reviews and is outside the scope of this review. A further reason not to undertake critical appraisal was the diversity of the grey literature considered for inclusion. However, the sources of information of the reviewed documents will be inspected, to provide insight on the credibility and accuracy of the advice provided.

For peer review only - http://bmjopen.bmj.com/site/about/guidelines.xhtml

For peer review only

Data Analysis and Presentation

The characteristics of included resources will be described in detail (e.g., participants, target audience), a distribution of studies by year will be produced and a map of the available evidence will be presented in tables. Comparisons will be made based on the included evidence across the UK and Ireland, and resources addressing preconception health and care services, interventions and initiatives in Northern Ireland will be presented as a case study. Included resources will be coded using NVivo, and at least 10% of the data will be double-coded. A narrative synthesis will highlight the emerging themes and concepts and emphasise how the findings relate to the research questions. The limitations and research gaps will be emphasised and discussed. Authors will adhere to PRISMA-ScR when reporting findings[15].

Patient and Public Involvement and Engagement

The proposed review actively involves a Patient and Public Involvement and Engagement (PPIE) panel of adults aged 18-45 years living in Northern Ireland, named “Healthy Reproductive Years”. The PPIE strategies aim to engage the public as partners and mobilise their existing knowledge and expertise, communicate using a lay language and support a collaborative approach in research by fostering respect and honesty[26]. The PPIE advisory panel was recruited via social media platforms (e.g., Facebook), relevant organisations (e.g., Sure Start), charities and leisure and community centres in Northern Ireland. Recruitment activities aimed to achieve diversity in terms of gender, sexuality, ethnicity, religion, disability, socioeconomic background, education level and health literacy. The PPIE panel was developed to contribute to general discussions and advise on research design, priority setting and dissemination plans which will include the publication of the final report and the co-production of conference materials, a lay summary and an infographic presenting review findings. Representatives have been integrally involved in the development of this protocol and have provided direct feedback on all aspects of it. Collaboration with the PPIE panel will occur via online or in-person meetings and exchange of emails or postal letters. The reporting of PPIE strategies in this research project will be guided by the Guidance for

For peer review only - http://bmjopen.bmj.com/site/about/guidelines.xhtml

Reporting Involvement of Patients and the Public (GRIPP) 2 checklist[27]. All PPIE representatives will be reimbursed for their contributions.

Ethics and dissemination:

Ethical approval is not applicable because this scoping review will analyse publicly accessible resources only. The consultations and audits with key stakeholders conducted to validate findings relative to Northern Ireland do not require ethical approval as these are only intended as tools to inform research. Findings will be disseminated to service users and relevant stakeholders using diverse approaches, targeted to recipients. Outcomes following this review include a peer-reviewed publication reporting a detailed synthesis of findings and comparisons across countries, a lay summary, a clear and accessible infographic, and presentations directed at conference audiences, stakeholders and the public. The PPIE "Healthy Reproductive Years" panel will be consulted and will inform the dissemination plan. Additionally, a workshop will be held with PPIE representatives to discuss findings.

Discussion:

Recent years have seen the rise in the publication of key documents addressing preconception health and care that have the opportunity to positively influence the current landscape by supporting positive health changes, including Making the Case for Preconception Care[2], Missed Periods[28], A Strategy for Maternity Care in Northern Ireland[23] and Women's Health Strategy for England[29]. These documents emphasise the need to build the evidence base, raise awareness on women's health and preconception health and care, improve information-sharing and ultimately ensure that all adults of reproductive years are given the tools to optimise their preconception physical, social and psychological health. Recently, it has also been announced that folic acid will be added to non-wholemeal wheat flour in the UK as a way to reduce the incidents of neural tube defects[25]. It is also important to acknowledge that efforts have been made globally. Examples include a policy brief published by the World Health Organization with the aim of improving preconception care[3] and the identification

For peer review only - http://bmjopen.bmj.com/site/about/guidelines.xhtml

of a condensed set of preconception health indicators in the United States with the aim of better monitoring the health status of women of reproductive age[1].

Currently, no comprehensive synthesis of the evidence relating to this topic is available in the proposed context of the UK and Ireland. Although this review has potential strengths, such as the transparency of the approach adopted, the addition of consultations with experts and the involvement of a PPIE advisory panel, there are also challenges and limitations, such as the broad scope of the review and the lack of a formal critical appraisal of the individual resources. Both strengths and limitations will be clearly explored and further highlighted in the full report.

For peer review only

For peer review only - http://bmjopen.bmj.com/site/about/guidelines.xhtml

References:

1. Robbins CL, D'Angelo D, Zapata L, et al. Preconception health indicators for public health surveillance. *J Womens Health (Larchmt)*2018;27:430-443. doi:10.1089/jwh.2017.6531
2. Public Health England. Making the case for preconception care. 2018.https://www.gov.uk/government/publications/preconception-care-making-the-case (accessed 22 Apr. 2022).
3. World Health Organization. Policy brief: preconception care – maximising the gains for maternal and child health. 2013.https://www.who.int/publications/i/item/WHO-FWC-MCA-13.02 (accessed 22 Apr. 2022).
4. Evans H, Buck D. Tackling multiple unhealthy risk factors. 2018.https://www.kingsfund.org.uk/sites/default/files/2018-03/Tackling%20multiple%20unhealthy%20risk%20factors%20-%20full%20report.pdf (accessed 22 Apr. 2022).
5. Broussard DL, Sappenfield WB, Fussman C, et al. Core state preconception health indicators: a voluntary, multi-state selection process. *Matern Child Health J*2011;15:158-68. doi:10.1007/s10995-010-0575-x
6. Schoenaker DAJM, Stephenson J, Connolly A, et al. Characterising and monitoring preconception health in England: a review of national population-level indicators and core data sources. *J Dev Orig Health Dis*2021;13:137-150. doi:10.1017/S2040174421000258
7. Public Health England. Maternity high impact area: Reducing the inequality of outcomes for women from Black, Asian and Minority Ethnic (BAME) communities and their babies. 2020.https://assets.publishing.service.gov.uk/government/uploads/system/uploads/attachment_data/file/942480/Maternity_high_impact_area_6_Reducing_the_inequality_of_outcomes_for_women_from_Black__Asian_and_Minority_Ethnic__BAME__communities_and_their_babies.pdf (accessed 26 July 2022).
8. Public Health England. Health of women before and during pregnancy: health behaviours, risk factors and inequalities. 2019.https://assets.publishing.service.gov.uk/government/uploads/system/uploads/

For peer review only - http://bmjopen.bmj.com/site/about/guidelines.xhtml

attachment_data/file/844210/Health_of_women_before_and_during_pregnancy_2019.pdf (accessed 22 Apr. 2022).

9. Shawe J, Delbaere I, Ekstrand M, et al. Preconception care policy, guidelines, recommendations and services across six European countries: Belgium (Flanders), Denmark, Italy, the Netherlands, Sweden and the United Kingdom. *Eur J Contracept Reprod Health Care*2015;20:77-87. doi:10.3109/13625187.2014.990088
10. Wellings K, Jones KG, Mercer CH, et al. The prevalence of unplanned pregnancy and associated factors in Britain: findings from the third National Survey of Sexual Attitudes and Lifestyles (Natsal-3). *Lancet*2013;382(9907):1807-1816. Doi: 10.1016/S0140-6736(13)62071-1.
11. Schoenaker DAJM, Stephenson J, Smith H, et al. Women's preconception health in England: a report card based on national maternity services data 2018/19. *Lancet* [Preprint]2022. http://dx.doi.org/10.2139/ssrn.4163702 (accessed 2 Aug. 2022).
12. E, Boyle JA, Walker R, et al. A systematic review of clinical guidelines for preconception care. *Semin Reprod Med*2022;40:157-169. doi:10.1055/s-0042-1748190
13. Arksey H, O'Malley L. Scoping studies: towards a methodological framework. *Int J Soc Res Methodol*2005;8:19-32. doi:10.1080/1364557032000119616
14. Peters MDJ, Marnie C, Tricco AC, et al. Updated methodological guidance for the conduct of scoping reviews. *JBI Evid Synth*2020;18:2119-2126. doi: 10.11124/JBIES-20-00167
15. Tricco AC, Lillie E, Zarin W, et al. PRISMA extension for scoping reviews (PRISMA-ScR): Checklist and Explanation. *Ann Intern Med*2018;169:467-473. doi:10.7326/M18-0850
16. Peters MDJ, Godfrey C, McInerney P, et al. *Chapter 11: Scoping Reviews*. In: Aromataris E, Munn Z (Editors). JBI Manual for evidence synthesis. Adelaide: The Joanna Briggs Institute 2020
17. Gagnon, YC. *The case study as a research method*. Canada: Presses de l'Universite du Quebec 2010.
18. Watson D, Jacob CM, Giles G, et al. A scoping review of nutritional interventions and policy guidelines in the interconception period for prevention of noncommunicable diseases. *Reprod Female Child Health*2022. doi:10.1002/rfc2.8

For peer review only - http://bmjopen.bmj.com/site/about/guidelines.xhtml

19. Pappas C, Williams I. Grey literature: its emerging importance. *J Hosp Librariansh*2011,11:228-234. Doi:10.1080/15323269.2011.587100
20. Aromataris E, Munn Z (Editors). *Chapter 2: Systematic reviews of qualitative evidence.* In: JBI Manual for Evidence Synthesis. *Adelaide:* JBI 2020. https://doi.org/10.46658/JBIMES-20-01
21. Paez A. Grey literature: An important resource in systematic reviews. *J Evid Based Med*. 2017,10:233-240. Doi: 10.1111/jebm.12265.
22. Godin K, Stapleton J, Kirkpatrick SI, et al. Applying systematic review search methods to the grey literature: a case study examining guidelines for school-based breakfast programs in Canada. *Syst Rev*2015;4:138. doi:10.1186/s13643-015-0125-0
23. Department of Health, Social Services and Public Safety. A strategy for maternity care in Northern Ireland. 2012.https://www.health-ni.gov.uk/sites/default/files/publications/dhssps/maternitystrategy.pdf (accessed 5 May 2022).
24. Department of Health. Mental Health Action Plan. 2020.https://www.health-ni.gov.uk/sites/default/files/publications/health/mh-action-plan-plus-covid-response-plan.pdf (accessed 16 February 2023).
25. UK Government. Proposal to add folic acid to flour: consultation response. 2021.https://www.gov.uk/government/consultations/adding-folic-acid-to-flour/outcome/proposal-to-add-folic-acid-to-flour-consultation-response (accessed 20 July 2022).
26. Joosten YA, Israel TL, Williams NA, et al. Community engagement studios: a structured approach to obtaining meaningful input from stakeholders to inform research. *Acad Med*2015;90:1646-50. doi:10.1097/ACM.0000000000000794
27. Staniszewska S, Brett J, Simera I, et al. GRIPP2 reporting checklists: tools to improve reporting of patient and public involvement in research. *Res Involv Engagem*2017;3:13. doi:10.1136/bmj.j3453
28. Sher J. Missed periods. 2016.https://www.nhsggc.org.uk/media/237840/missed-periods-j-sher-may-2016.pdf (accessed 5 May 2022).
29. Department of Health & Social Care. Women's Health Strategy for England. 2022.https://www.gov.uk/government/publications/womens-health-strategy-for-england/womens-health-strategy-for-england (accessed 26 July 2022).

For peer review only - http://bmjopen.bmj.com/site/about/guidelines.xhtml

Authors' Contributions:

EC, LM, MM, LK discussed the protocol and finalised the methodological process of the scoping review. EC drafted the protocol, which was finalised with input from LM, MM, LK, KAE and DS. DT and TS, members of the Patient and Public Involvement and Engagement "Healthy Reproductive Years" panel, also helped shape the research and reviewed the protocol. All authors have seen and approved the final version of the protocol for publication.

Acknowledgements:

The authors would like to acknowledge the contribution of the Patient and Public Involvement and Engagement "Healthy Reproductive Years" panel for their assistance.

Funding Statement:

This work is supported by Queen's University Belfast as part of a PhD studentship for EC. EC is funded by the Department for the Economy NI. DS is supported by the National Institute for Health and Social Care Research (NIHR) Southampton Biomedical Research Centre [IS-BRC-1215-20004]). The views expressed are those of the author(s) and not necessarily those of the NIHR or the Department of Health and Social Care.

Competing Interests Statement:

There is no conflict of interest in this project.

Keywords:

Pre-conception; reproductive health; pregnancy; grey literature.

For peer review only

Appendices:

Appendix I: Preconception risk factors

**Social**		
Adverse childhood experiences<br>Belonging to an ethnic minority<br>Deprivation<br>Domestic abuse (physical and emotional)	Environmental tobacco smoke and second-hand smoke exposure<br>Exposure to toxic or hazardous substances<br>Financial insecurity or unemployment<br>Inadequate support from family or social network	Low educational attainment<br>Migration status<br>Unsuitable access to quality healthcare and dentalcare<br>Unsuitable housing
**Behavioural**		
Alcohol misuse<br>Eating disorders<br>Inadequate dietary habits	Lack of family planning/pregnancy intention<br>Physical inactivity or sedentary behaviours	Smoking<br>Substance misuse<br>Use of teratogenic medications
**Biomedical**		
Fertility issues<br>Folic acid or other vitamin deficiency<br>General health status<br>Genetic risks<br>Infections (e.g., sexually transmitted diseases)	Low immunisation levels<br>Mental health conditions (e.g., anxiety, depression)<br>Parental age<br>Parental obesity or overweight, parental underweight	Physical health conditions (e.g., diabetes mellitus, epilepsy, endometriosis, chronic hypertension)<br>Previous pregnancy and obstetric complications (e.g., previous foetal death, miscarriage, stillbirth, preterm birth)

References for the table: [2,5,6]

For peer review only - http://bmjopen.bmj.com/site/about/guidelines.xhtml

Appendix II: Glossary of terms.

Term	Definition
Preconception health	The overall health of non-pregnant individuals of childbearing age[1].
Preconception care	The "services, interventions, support and advice" aimed at optimising pregnancy planning and fitness for pregnancy[2].
Interconception	Period between the end of a pregnancy and the start of the next[29].

For peer review only

For peer review only - http://bmjopen.bmj.com/site/about/guidelines.xhtml

Appendix III: Inclusion and exclusion criteria for a scoping review investigating strategies, policies, services, guidelines, frameworks and recommendations that address preconception health and care for adults in the UK and Ireland.

**Inclusion**	**Exclusion**
The resource is one of the following: strategies, policies, guidelines, frameworks and recommendations, including technical or research reports from government agencies, registered charities or scientific research groups, documents outlining interventions or initiatives from public bodies, articles and guidelines issued by government agencies, Royal Colleges and professional bodies, e-learning resources, leaflets and educational booklets. If the resource is a service, intervention or initiative it will be included only if pertinent to Northern Ireland.	The resource is one of the following: journal articles, preprints (journal articles not yet peer-reviewed or published), working papers from research groups, visual or audio content (or reviews of visual or audio content), academic letters or commentaries, calls for participants, presentations and doctoral dissertations. If the resource is a service, intervention or initiative it will be excluded if not pertinent to Northern Ireland.
The resource addresses preconception health and care.	The resource does not address preconception health and care. This includes if the resource explicitly addresses sexual health, interconception health and care or pregnancy.
The resource is relevant to the UK or Ireland.	The resource is not relevant to the UK or Ireland.
The resource was published, reviewed or updated during or after January 2011.	The resource was published, reviewed or updated before January 2011.
The resource was published in English.	The resource was published in any language other than English.
The resource presents unique insights and does not simply report, cite or signpost other included resources.	The resource mentions preconception health and care only by reporting, citing or signposting included resources, and therefore duplicates findings of an earlier version of the resource.
The resource is currently available.	The resource is not currently available (e.g., guideline being updated).

For peer review only

Appendix VI: Search strategy

List of searches conducted on Advanced Google Search for Northern Ireland.

	**Search terms**
1	~preconception AND "Northern Ireland" OR NI
2	~preconception AND "Northern Ireland" AND policy OR service OR intervention OR guideline OR strategy OR initiative
3	"preconception health" OR "preconception care" AND "Northern Ireland" OR NI
4	~pre-pregnancy AND "Northern Ireland" OR NI AND policy OR service OR intervention OR guideline OR strategy OR initiative
5	~preconception AND "Northern Ireland" OR NI AND ~charity
6	~preconception AND "Health and Social Care Trust" OR HSCT
7	"pregnancy planning" OR "planning a baby" OR "preparation for pregnancy" OR "preparation for parenthood" OR "pregnancy intention" OR "expecting mothers" OR "expectant mothers" OR "conceive" OR "future pregnancy" AND "Northern Ireland"
8	"reproductive health" OR "sexual health" AND "Northern Ireland" AND policy OR service OR intervention OR guideline OR strategy OR initiative

Results limited based on language (English) and country (United Kingdom).

For peer review only

For peer review only - http://bmjopen.bmj.com/site/about/guidelines.xhtml

Appendix V: Additional intended sources and websites

Source	Website	Context
National Institute for Health Research	https://www.nihr.ac.uk/	UK
British National Health Service (NHS)	https://www.nhs.uk/	UK
Royal College of Obstetricians and Gynaecologists	https://www.rcog.org.uk/	UK
Tommy's	https://www.tommys.org/	England, Scotland, Wales
Office for Health Improvement & Disparities	https://www.gov.uk/government/organisations/office-for-health-improvement-and-disparities	England
Public Health Wales	https://phw.nhs.wales/	Wales
Public Health Scotland	https://www.publichealthscotland.scot/	Scotland
NHS Inform	https://www.nhsinform.scot/	Scotland
Health Well	https://healthwell.eani.org.uk	Northern Ireland
Department of Health (Northern Ireland)	https://www.health-ni.gov.uk/	Northern Ireland
Sexual Health NI	https://www.sexualhealthni.info/	Northern Ireland
NI DIRECT	https://www.nidirect.gov.uk/	Northern Ireland
Public Health Agency	https://www.publichealth.hscni.net/	Northern Ireland
Health Service Executive IE	https://www.hse.ie/eng/	Ireland
Health Information and Quality Authority	https://www.hiqa.ie/areas-we-work/health-information/data-collections/all-ireland-public-health-repository	Ireland

For peer review only - http://bmjopen.bmj.com/site/about/guidelines.xhtml

Appendix VI: Example of completed data extraction instrument.

Reference #	Reviewed by	Title & url	Resource format	Source	Date accessed	Date published/last updated or modified	Duration, when applicable	Context	Notes/Short description	Key themes
63	EHC, LM	Hypertension in pregnancy - Quality standard [QS35] > https://www.nice.org.uk/guidance/qs35	Guideline	NICE	02-May-22	2019	NA	UK	"Women of childbearing potential with treated hypertension are given information annually about safe antihypertensive treatment during pregnancy." "Information can be provided to women who may become pregnant about safe antihypertensive treatment during pregnancy as part of an annual review of hypertension care. Women should be informed about potential risks, including the risk of congenital abnormalities, linked to particular antihypertensive drugs. This should enable women to arrange a discussion with the healthcare professional responsible for managing their hypertension about alternative antihypertensive treatments if they are planning pregnancy or become pregnant." (excerpts)	Blood pressure, hypertension, long-term conditions, Preconception advice

For peer review only - http://bmjopen.bmj.com/site/about/guidelines.xhtml

Appendix VII: Revised data extraction instrument.

**Data to be extracted**	**Notes to reviewer**
Reference #	
Reviewed by	
Title	
Resource format	e.g., report, intervention, strategy, policy
Source	e.g., Google Advanced Search, NICE, OpenAire
Date accessed	
Year of publication/Year of most recent update.	
Duration	When applicable, the duration of the strategy, policy or intervention will be reported
Participants*	Target population
Context	Country
Target audience*	e.g., healthcare professionals, policymakers
Overarching aim*	e.g., educate, increase awareness
Key notes on topics/content	

* Added following piloting phase.

For peer review only

Preferred Reporting Items for Systematic reviews and Meta-Analyses extension for Scoping Reviews (PRISMA-ScR) Checklist

SECTION	ITEM	PRISMA-ScR CHECKLIST ITEM	REPORTED ON PAGE #
**TITLE**			
Title	1	Identify the report as a scoping review.	i
**ABSTRACT**			
Structured summary	2	Provide a structured summary that includes (as applicable): background, objectives, eligibility criteria, sources of evidence, charting methods, results, and conclusions that relate to the review questions and objectives.	ii
**INTRODUCTION**			
Rationale	3	Describe the rationale for the review in the context of what is already known. Explain why the review questions/objectives lend themselves to a scoping review approach.	1-3
Objectives	4	Provide an explicit statement of the questions and objectives being addressed with reference to their key elements (e.g., population or participants, concepts, and context) or other relevant key elements used to conceptualize the review questions and/or objectives.	3-4
**METHODS**			
Protocol and registration	5	Indicate whether a review protocol exists; state if and where it can be accessed (e.g., a Web address); and if available, provide registration information, including the registration number.	NA
Eligibility criteria	6	Specify characteristics of the sources of evidence used as eligibility criteria (e.g., years considered, language, and publication status), and provide a rationale.	5-6
Information sources*	7	Describe all information sources in the search (e.g., databases with dates of coverage and contact with authors to identify additional sources), as well as the date the most recent search was executed.	7
Search	8	Present the full electronic search strategy for at least 1 database, including any limits used, such that it could be repeated.	Supplementary Material
Selection of sources of evidence†	9	State the process for selecting sources of evidence (i.e., screening and eligibility) included in the scoping review.	8-9
Data charting process‡	10	Describe the methods of charting data from the included sources of evidence (e.g., calibrated forms or forms that have been tested by the team before their use, and whether data charting was done independently or in duplicate) and any processes for obtaining and confirming data from investigators.	9-10
Data items	11	List and define all variables for which data were sought and any assumptions and simplifications made.	NA
Critical appraisal of individual sources of evidence§	12	If done, provide a rationale for conducting a critical appraisal of included sources of evidence; describe the methods used and how this information was used in any data synthesis (if appropriate).	NA
Synthesis of results	13	Describe the methods of handling and summarizing the data that were charted.	9-10

For peer review only - http://bmjopen.bmj.com/site/about/guidelines.xhtml

SECTION	ITEM	PRISMA-ScR CHECKLIST ITEM	REPORTED ON PAGE #
**RESULTS**			
Selection of sources of evidence	14	Give numbers of sources of evidence screened, assessed for eligibility, and included in the review, with reasons for exclusions at each stage, ideally using a flow diagram.	NA
Characteristics of sources of evidence	15	For each source of evidence, present characteristics for which data were charted and provide the citations.	NA
Critical appraisal within sources of evidence	16	If done, present data on critical appraisal of included sources of evidence (see item 12).	NA
Results of individual sources of evidence	17	For each included source of evidence, present the relevant data that were charted that relate to the review questions and objectives.	NA
Synthesis of results	18	Summarize and/or present the charting results as they relate to the review questions and objectives.	NA
**DISCUSSION**			
Summary of evidence	19	Summarize the main results (including an overview of concepts, themes, and types of evidence available), link to the review questions and objectives, and consider the relevance to key groups.	NA
Limitations	20	Discuss the limitations of the scoping review process.	NA
Conclusions	21	Provide a general interpretation of the results with respect to the review questions and objectives, as well as potential implications and/or next steps.	NA
**FUNDING**			
Funding	22	Describe sources of funding for the included sources of evidence, as well as sources of funding for the scoping review. Describe the role of the funders of the scoping review.	16

JBI = Joanna Briggs Institute; PRISMA-ScR = Preferred Reporting Items for Systematic reviews and Meta-Analyses extension for Scoping Reviews.

* Where *sources of evidence* (see second footnote) are compiled from, such as bibliographic databases, social media platforms, and Web sites.

† A more inclusive/heterogeneous term used to account for the different types of evidence or data sources (e.g., quantitative and/or qualitative research, expert opinion, and policy documents) that may be eligible in a scoping review as opposed to only studies. This is not to be confused with *information sources* (see first footnote).

‡ The frameworks by Arksey and O'Malley (6) and Levac and colleagues (7) and the JBI guidance (4, 5) refer to the process of data extraction in a scoping review as data charting.

§ The process of systematically examining research evidence to assess its validity, results, and relevance before using it to inform a decision. This term is used for items 12 and 19 instead of "risk of bias" (which is more applicable to systematic reviews of interventions) to include and acknowledge the various sources of evidence that may be used in a scoping review (e.g., quantitative and/or qualitative research, expert opinion, and policy document).

From: Tricco AC, Lillie E, Zarin W, O'Brien KK, Colquhoun H, Levac D, et al. PRISMA Extension for Scoping Reviews (PRISMAScR): Checklist and Explanation. Ann Intern Med. 2018;169:467–473. doi: 10.7326/M18-0850.

For peer review only - http://bmjopen.bmj.com/site/about/guidelines.xhtml

BMJ Open

Preconception health and care policies, strategies and guidelines in the UK and Ireland: A scoping review protocol

Journal:	*BMJ Open*
Manuscript ID	bmjopen-2022-067822.R2
Article Type:	Protocol
Date Submitted by the Author:	07-Apr-2023
Complete List of Authors:	Cassinelli, Emma ; Queen's University Belfast,<br>McKinley, Michelle C.; Queen's University Belfast<br>Kent, Lisa; Queen's University Belfast, Centre for Public Health<br>Eastwood, Kelly-Ann; University Hospitals Bristol and Weston NHS Foundation Trust; Queen's University Belfast<br>Schoenaker , Danielle; University of Southampton,<br>Trew, David; Patient and Public Involvement and Engagement<br>Stoikidou, Theano ; Patient and Public Involvement and Engagement<br>McGowan, Laura; Queen's University Belfast
**Primary Subject Heading**:	Public health
Secondary Subject Heading:	Health policy, Sexual health
Keywords:	PUBLIC HEALTH, Maternal medicine < OBSTETRICS, Reproductive medicine < GYNAECOLOGY, Protocols & guidelines < HEALTH SERVICES ADMINISTRATION & MANAGEMENT

SCHOLARONE™ Manuscripts

Title: Preconception health and care policies, strategies and guidelines in the UK and Ireland: A scoping review protocol

Authors: Emma Hope Cassinelli[1], Prof Michelle McKinley[1], Dr Lisa Kent[1], Dr Kelly-Ann Eastwood [1,2], Dr Danielle Schoenaker[3], David Trew[4], Theano Stoikidou[4], Dr Laura McGowan[1]

1. Centre for Public Health (Institute for Global Food Security), School of Medicine, Dentistry and Biomedical Sciences, Queen's University Belfast, Belfast (United Kingdom).

2. University Hospitals Bristol NHS Foundation Trust, Bristol (United Kingdom).

3. NIHR Southampton Biomedical Research Centre, University of Southampton and University Hospital Southampton NHS Foundation Trust, Southampton (United Kingdom).

4 Patient and Public Involvement and Engagement "Healthy Reproductive Years" Panel

Corresponding author: Dr Laura McGowan, Lecturer in Nutrition and Behaviour Change.

Postal address: Institute for Global Food Security (Centre for Public Health), Queen's University Belfast, Institute of Clinical Sciences, Block A. Grosvenor Road, Belfast, BT12 6BJ, United Kingdom.

Email address: laura.mcgowan@qub.ac.uk

For peer review only

Abstract:

Introduction: Preconception health and care strategies can significantly improve maternal and infant outcomes, and thus optimise intergenerational health. The aims of this scoping review are to 1) provide an up-to-date summary of preconception health and care strategies, policies, guidelines, frameworks and recommendations across the UK and Ireland, and 2) explore preconception health and care services, interventions and initiatives in Northern Ireland as a case study.

Methods and analysis: The scoping review of grey literature will be conducted as per the Scoping Review Methods Manual by the Joanna Briggs Institute and the Arksey-O'Malley framework for scoping studies, and reported in line with PRISMA-ScR. Searches were conducted on Google Advanced Search, OpenAire, NICE, ProQuest and relevant public health websites in May 2022. Only results published, reviewed, or updated between January 2011 and the time of the search (May 2022) were considered for inclusion. Additionally, searches on interventions, services and initiatives provided in Northern Ireland will be supplemented by consultations and audits with key stakeholders to validate findings, identify other potentially eligible resources and ensure breadth of coverage. Data will be extracted into Excel and coded using NVivo, and ≥10% of the data will be double-coded. A narrative approach with content analysis highlighting key themes and concepts will be used to report findings.

Throughout the research cycle, members of the wider public will be involved and engaged with to provide feedback.

Ethics and dissemination: Ethical approval is not required as analyses will be conducted on data available in the public domain. Findings will be shared with relevant stakeholders with the aim to inform future research, practice and decision-making, and disseminated through a peer-reviewed publication, conference presentations and infographics. Dissemination plans will be informed by the “Healthy Reproductive years” PPIE advisory panel.

For peer review only - http://bmjopen.bmj.com/site/about/guidelines.xhtml

Strengths and limitations of this study:

1. This scoping review will be conducted following a systematic process to transparently locate and synthesise evidence, informed by the Joanna Briggs Institute updated methodological guidance and Arksey and O'Malley's framework, and will be reported in line with the Preferred Reporting Items for Systematic Reviews and Meta-analyses extension for scoping reviews (PRISMA-ScR).
2. This review will incorporate contributions and feedback from those with lived experiences of the preconception period throughout.
3. Consultations and audits will be undertaken to validate findings relevant to Northern Ireland, as a case study, identify other potentially eligible resources and understand any under-researched issues concerning the effectiveness of preconception care services or strategies.
4. No quality appraisal will be conducted on the included material, as this is a scoping review aiming to map the breadth of information on the chosen topic.

For peer review only

For peer review only - http://bmjopen.bmj.com/site/about/guidelines.xhtml

Introduction:

Preconception health describes the overall health of non-pregnant individuals of childbearing age, which usually defines individuals aged 18-44 years[1]. The optimisation of preconception health can significantly improve maternal and infant outcomes and, therefore, represents a window of opportunity to improve the health of future generations[2]. Preconception care is the term used to define biomedical, behavioural and social health interventions, services, support and advice provided prior to conception aimed at optimising pregnancy planning and fitness for pregnancy[2,3].

The health status of individuals in the preconception stage can be negatively impacted by risk factors that are often modifiable and/or reversible, and people often have more than one risk factor concurrently[4]. Preconception health risk factors include, for example, parental obesity, long-term physical and mental health conditions, alcohol consumption, smoking, physical inactivity, inadequate dietary habits, low folic acid intake, poor social support, emotional ill-health and low immunisation levels[2,5,6]. While some risk factors are modifiable, such as folic acid intake, others may require further evidence-based support and long-term management to achieve and sustain change, such as weight management in people living with obesity. Risk factors such as socioeconomic status are not easily modifiable and, therefore, demand efforts targeted at vulnerable groups of people affected by inequalities[7]. For example, compared to women from the least deprived populations, investigations in England have shown that women living in more deprived areas may not only be more likely to be overweight or living with obesity and report the misuse of illicit drugs and smoking, but they may also be less likely to stop smoking during pregnancy, take folic acid supplements and book their first antenatal appointment within 10 weeks of pregnancy as recommended[8]. A comprehensive list of preconception health risk factors is provided in Appendix I.

Preconception care plays a crucial role in the screening, prevention and management of risk factors that may affect maternal and infant outcomes[9]. The provision of preconception care includes the development and implementation of strategies, policies, services, interventions, campaigns, guidelines, frameworks and initiatives. Because preconception

For peer review only - http://bmjopen.bmj.com/site/about/guidelines.xhtml

care operates at individual, community and population levels, it can positively impact both those who actively plan to conceive and those who do not. This is of particular interest because of the high prevalence of unplanned pregnancies; in the UK, 45% of pregnancies and one-third of births are unplanned or accompanied by feelings of ambivalence[2, 10].

Recent years have witnessed an increased recognition of the importance of preconception health and care[6], in both the academic field and policy (e.g., Making the Case for Preconception Care[2]), suggesting that recent publicly accessible resources may have been produced but not yet reviewed in this capacity.

Shawe and colleagues conducted an investigation of preconception policies, guidelines, recommendations and services available in six different countries, including the UK, to provide a baseline comparison of current materials [9]. They found variations between recommendations (e.g., folic acid supplementation) and concluded that guidelines addressing the preconception phase are overall heterogeneous[9]. As these previous findings are related to searches conducted in 2013, a renewed investigation is now warranted covering the past decade.

A further study on preconception guidelines, recommendations and policy reports was conducted in 2021[6]. The review, however, intended to inform the reporting of population-level preconception health in England and identify preconception indicators, which then formed the basis for the development of a national surveillance system for preconception health in England[11]. Sixty-six indicators were identified across 12 domains, namely wider determinants of health, health care, environmental exposures, reproductive health and family planning, health behaviours, cervical screening, immunisation and infections, mental and physical health, medications and genetic risk[6].

A systematic review was recently conducted to analyse freely accessible international clinical guidelines focusing on preconception care[12]. They identified eleven guidelines and assessed the quality of the guidelines themselves using the AGREE II tool, the overall recommendations and the evidence recommendations are based on[12]. Findings suggested that high-quality guidelines are lacking and that guidelines should be improved by broadening the comprehensiveness of the content areas addressed, applying a more

For peer review only - http://bmjopen.bmj.com/site/about/guidelines.xhtml

rigorous development process and enhancing the acceptability and feasibility of their application[12].

No previous review was found that included both proposed locations of this scoping review: UK and Ireland.

Objectives

From the available literature it is clear that at present it is important to collate the evidence in a coherent and accessible way that enables the identification of common themes, concepts and recommendations, priority areas not yet attended to and strengths and weaknesses of preconception care actions in place across the UK and Ireland. This scoping review aims to build upon existing research and map the evidence in relation to preconception health and care strategies, policies, guidelines, frameworks and recommendations in the UK and Ireland, by summarising recurring themes and concepts underpinning the evidence, identifying gaps in knowledge and exploring future research priorities. This review will also clarify the type of evidence available and the target audience/s of included resources. Northern Ireland will be treated as a case study and, therefore, data on the services, interventions and initiatives provided in the region will be examined, presented and supplemented by consultations and audits with relevant stakeholders.

A systematic scoping approach will be used, as it is appropriate to map the principal themes of a broad research area of interest, describe the breadth of evidence available and synthesise heterogeneous evidence[13]. The present protocol will ensure transparency in the research process and overall reliability[14].

Findings from this review will ultimately inform future research, practice and decision-making to optimise preconception health and care, and may help the development of a clear pathway for the promotion of evidence-based advice and support for individuals of childbearing age. Additionally, findings may aid the planning of preconception care actions, potentially leveraging and maximising existing public health programmes.

For peer review only - http://bmjopen.bmj.com/site/about/guidelines.xhtml

For peer review only

Research Questions

This scoping review aims to answer the following questions:

a. What strategies, policies, guidelines, frameworks and recommendations have been developed that address preconception health and care for adults in the UK and Ireland between January 2011 and May 2022?
b. What are the main concepts and themes underpinning strategies, policies, guidelines, frameworks and recommendations that address preconception health and care for adults in the UK and Ireland?
c. How does the evidence from strategies, policies, guidelines, frameworks and recommendations that address preconception care for adults differ across the UK and Ireland?
d. What are, if any, the gaps in the knowledge provided in strategies, policies, services, guidelines, frameworks and recommendations that address preconception health and care for adults in the UK and Ireland, and what areas require further coverage and inquiry?
e. What are, if any, the services, initiatives and interventions provided in Northern Ireland focused on improving preconception health and care in adults?

Answering these questions will provide insights into how the topic of preconception health and care is addressed in the grey literature, summarise the evidence and present an overview of how evidence may differ across the selected countries.

Methods and analysis:

The proposed scoping review will be conducted in accordance with the Joanna Briggs Institute (JBI) updated methodological guidance for scoping reviews[14], Arksey and O'Malley's framework for conducting scoping studies[13] and reported in line with the Preferred Reporting Items for Systematic Reviews and Meta-analyses extension for scoping reviews (PRISMA-ScR)[15].

For peer review only - http://bmjopen.bmj.com/site/about/guidelines.xhtml

A preliminary search of CINAHL, Web of Science, JBI Evidence Synthesis and BMJ Open was conducted and no current or underway scoping reviews on the topic were identified.

Eligibility Criteria

The recent growing interest in preconception health and attention paid to the optimisation of health before pregnancy has led to an increase in preconception care actions by governments and public health organisations (e.g., folic acid fortification), meaning that there is evidence to meet the inclusion criteria for this scoping review.
To develop the eligibility criteria, the Participants-Concept-Context framework outlined by Peters and colleagues was used[16], as described below. These criteria were developed in accordance with the study objectives and informed by the meaningful findings highlighted by Schoenaker and colleagues[6] and the critical discourse presented in resources such as Making the Case for Preconception Care[2].

Participants: This review will consider resources discussing or reporting on preconception health and care for individuals of childbearing age, and no exclusions in relation to gender, ethnicity, culture, sexuality, health condition/s or disability status will be applied. Therefore, resources addressing individuals with chronic diseases, such as diabetes and epilepsy, will be considered for inclusion. Relevant resources adopting a life-course approach, thus addressing society as a whole, will be considered for inclusion as they also encompass individuals of childbearing age.

Concept: The present work will summarise evidence underpinning strategies, policies, guidelines, frameworks and recommendations in the UK and Ireland regarding preconception health and care (see Appendix II Glossary of terms), including topics such as fitness for pregnancy, pregnancy planning and preparation for pregnancy. Furthermore, it will review services, interventions and initiatives in Northern Ireland, as an individual case study. A case study approach allows an in-depth analysis of phenomena in a given context[17].

For peer review only - http://bmjopen.bmj.com/site/about/guidelines.xhtml

Resources explicitly addressing only the interconception period (see Appendix II Glossary of terms) will not be considered eligible, also to avoid duplication of research due to a recent policy review covering this specific period[18].

Context: The review will only include resources relevant to the UK and Ireland. Whenever it is unclear whether a resource is relevant, the author/s or relevant organisation/s will be contacted.

Types of Sources

This scoping review will consider grey literature sources not found in the published literature. In this case, the grey literature included refers to strategies, policies, guidelines, frameworks and recommendations addressing preconception health and care in the UK and Ireland. Technical or research reports from government agencies, registered charities or scientific research groups, documents outlining interventions or initiatives from public bodies, articles and guidelines issued by government agencies or professional bodies will be considered for inclusion. In addition, leaflets and educational booklets will be considered for inclusion, as well as relevant e-learning resources due to their increasing popularity among healthcare professionals and ability to provide information that is easily understandable. Journal articles, preprints (journal articles not yet peer-reviewed or published), working papers from research groups, visual or audio content such as television programmes or documentaries (or reviews of this content), academic letters or commentaries, calls for participants, presentations and doctoral dissertations will not be considered for inclusion in this scoping review.

Grey literature, including but limited to policies, was deemed of interest as opposed to published literature as it is directed at the public, patients, healthcare professionals and governments, who are the ones who may act directly as a response to recommendations in the documents to review[6]. Grey literature holds the potential to disseminate findings to wider audiences as it is largely accessible also to non-specialist audiences[19,20], and can provide a current, balanced and comprehensive view of the evidence[21].

For peer review only - http://bmjopen.bmj.com/site/about/guidelines.xhtml

Overall, to be considered for inclusion, the material will need to provide concrete, tangible advice and guidance, deliver recommendations or alternatively outline policy actions or strategic plans to improve preconception health and care for adults of childbearing age. Whenever a resource solely mentions preconception health or simply signposts other material, it will not be eligible for inclusion. Although this review aims to summarise grey literature, certain strategies, policies, guidelines, frameworks and recommendations identified during the searches may also be published in the literature.

Details of the inclusion and exclusion criteria applied is presented in Appendix III.

Search Strategy

Initial searches were undertaken on Google Advanced Search in March and April 2022 to develop the search strategy for database searches. A subject librarian was also consulted to build a transparent and robustly structured search strategy (see Appendix IV).

The search strategy was then applied in May 2022 using the databases Google Advanced Search, National Institute for Health & Clinical Excellence (NICE), OpenAire and ProQuest. Public health and government websites were also searched (see Appendix V). This process of locating sources was informed by Godin *et al*.[22]. Other grey literature databases such as EThOS, DART, Grey Literature Report, OpenDoar were explored but excluded as not deemed relevant for the study. Boolean operators OR and AND were used. The search strategy, including all identified keywords and index terms, was adapted for each database and/or information source. The reference list of included sources of evidence will be screened for additional sources. Consultations with experts will be conducted in Spring 2023 to inform research and contextualise findings. Therefore, they will be conducted once preliminary findings from the scoping review are collated.

Only documents published in English will be included, as the inquiry is limited to primarily English-speaking countries. Resources published, reviewed or updated during or after January 2011 will be considered for inclusion, building upon the timeframe of the previous review across six European countries[9], thus allowing for more than a decade of content to be considered. Material from UK- and/or Ireland-based Royal Colleges and charitable organisations and associations with a location or contact address in one of the devolved

For peer review only - http://bmjopen.bmj.com/site/about/guidelines.xhtml

nations will be deemed eligible for inclusion. Whenever a potentially relevant resource is not readily available or access is restricted, the relevant body or authors will be contacted.

Case study

Consultations and audits with relevant stakeholders are favourable to validate findings and ensure breadth of coverage[13]. Therefore, the searches specific to Northern Ireland will be supplemented by audits with stakeholders, including experts in the field of preconception and healthcare professionals (e.g., maternity service providers and clinical leads, consultant midwives, pharmacists). Northern Ireland was deemed of interest as a case study because, although part of the UK, its devolved government has the ability to set its own policies, legislations and agenda. Moreover, preconception health is increasingly receiving importance in Northern Ireland, which has led to the development of a Strategy for maternity care[23], increased funding for the development of perinatal mental health teams across Health and Social Care Trusts[24] and support for the introduction of the mandatory fortification of flour with folic acid, for example[25].

Overall, these consultations aim to identify other potentially eligible resources, understand any under-researched issues concerning the effectiveness of services or strategies and contextualise any findings. Stakeholders will be sent information about the project prior to a consultation and will be asked to comment on preliminary findings and on whether any relevant resources may be missing. Questions and topic areas discussed during audits will be informed by the findings of the searches conducted. Contributors will also receive communications about the findings of the study, once completed[26].

Selection of Sources of Evidence

Citations identified through searching will be collated and uploaded into Microsoft Excel and duplicates will be removed. Titles and summaries will then be screened by two independent reviewers for assessment against the inclusion criteria for the review. Following the methodology used by Godin and colleagues for examining grey literature[22], only the first 100 results on Google Advanced Search will be reviewed for potentially relevant titles. Potentially relevant sources will be retrieved in full, and the full texts will be assessed in

For peer review only - http://bmjopen.bmj.com/site/about/guidelines.xhtml

detail against the inclusion criteria by two independent reviewers. At least 10% of resources will be double-coded. Any disagreements that arise between the reviewers at each stage of the selection process will be resolved through discussion, or with an additional reviewer/s, until consensus is reached. Reasons for the exclusion of full-text sources of evidence that do not meet the inclusion criteria will be recorded and reported in the scoping review. Overall, the results of the search and the study inclusion process will be reported in full in the final scoping review and presented in a PRISMA-ScR flow diagram[15]. The diagram will illustrate where citations were discarded during the screening process, and a rationale for the exclusion of resources during the full-text screening will also be provided.

Data Extraction

Data will be extracted by the same authors who performed the screening and full-text review to ensure consistency. Data will be extracted from eligible resources independently by two reviewers using a data extraction tool developed by the reviewers on Excel. The data extracted will include specific details about the publication title, publication format (e.g., report, guideline, strategy), target audience (e.g., healthcare professionals, policymakers), overarching aim (e.g., increase awareness among individuals of childbearing age, educate healthcare professionals), participants (e.g., individuals of childbearing age, individuals of childbearing age with diabetes), geographic location, year of publication, review or updates, duration of the strategy, policy or intervention when applicable, and key notes relevant to the research questions. These extracted values were agreed upon after pilot testing the draft extraction form between authors (initial example shown in Appendix VI). The revised data extraction instrument (Appendix VII) will be amended if necessary during the data extraction process. The modifications will be detailed in the full scoping review. Any disagreements between the reviewers will be resolved through discussion, or with an additional reviewer/s. When required, an attempt will be made to contact the authors of original resources for clarification if data is unclear, or to request missing or additional data. A follow-up email will be sent after two weeks from the initial contact if no response is received. Thereafter, the missing or unclear data will be documented in the review as such. A critical appraisal of individual sources of evidence will not be undertaken, as this is not a requirement for scoping reviews and is outside the scope of this review. A further reason

For peer review only - http://bmjopen.bmj.com/site/about/guidelines.xhtml

not to undertake critical appraisal was the diversity of the grey literature considered for inclusion. However, the sources of information of the reviewed documents will be inspected, to provide insight on the credibility and accuracy of the advice provided.

Data Analysis and Presentation

The characteristics of included resources will be described in detail (e.g., participants, target audience), a distribution of studies by year will be produced and a map of the available evidence will be presented in tables. Comparisons will be made based on the included evidence across the UK and Ireland, and resources addressing preconception health and care services, interventions and initiatives in Northern Ireland will be presented as a case study. Included resources will be coded using NVivo, and at least 10% of the data will be double-coded. A narrative synthesis will highlight the emerging themes and concepts and emphasise how the findings relate to the research questions. The limitations and research gaps will be emphasised and discussed. Authors will adhere to PRISMA-ScR when reporting findings[15].

Patient and Public Involvement and Engagement

The proposed review actively involves a Patient and Public Involvement and Engagement (PPIE) panel of adults aged 18-45 years living in Northern Ireland, named “Healthy Reproductive Years”. The PPIE strategies aim to engage the public as partners and mobilise their existing knowledge and expertise, communicate using a lay language and support a collaborative approach in research by fostering respect and honesty[26]. The PPIE advisory panel was recruited via social media platforms (e.g., Facebook), relevant organisations (e.g., Sure Start), charities and leisure and community centres in Northern Ireland. Recruitment activities aimed to achieve diversity in terms of gender, sexuality, ethnicity, religion, disability, socioeconomic background, education level and health literacy. The PPIE panel was developed to contribute to general discussions and advise on research design, priority setting and dissemination plans which will include the publication of the final report and the co-production of conference materials, a lay summary and an infographic presenting review findings. Representatives have been integrally involved in the development of this protocol

For peer review only - http://bmjopen.bmj.com/site/about/guidelines.xhtml

and have provided direct feedback on all aspects of it. Collaboration with the PPIE panel will occur via online or in-person meetings and exchange of emails or postal letters.

The reporting of PPIE strategies in this research project will be guided by the Guidance for Reporting Involvement of Patients and the Public (GRIPP) 2 checklist[27]. All PPIE representatives will be reimbursed for their contributions.

Ethics and dissemination:

Ethical approval is not applicable because this scoping review will analyse publicly accessible resources only. The consultations and audits with key stakeholders conducted to validate findings relative to Northern Ireland do not require ethical approval as these are only intended as tools to inform research. Findings will be disseminated to service users and relevant stakeholders using diverse approaches, targeted to recipients. Outcomes following this review include a peer-reviewed publication reporting a detailed synthesis of findings and comparisons across countries, a lay summary, a clear and accessible infographic, and presentations directed at conference audiences, stakeholders and the public. The PPIE "Healthy Reproductive Years" panel will be consulted and will inform the dissemination plan. Additionally, a workshop will be held with PPIE representatives to discuss findings.

Discussion:

Recent years have seen the rise in the publication of key documents addressing preconception health and care that have the opportunity to positively influence the current landscape by supporting positive health changes, including Making the Case for Preconception Care[2], Missed Periods[28], A Strategy for Maternity Care in Northern Ireland[23] and Women's Health Strategy for England[29]. These documents emphasise the need to build the evidence base, raise awareness on women's health and preconception health and care, improve information-sharing and ultimately ensure that all adults of reproductive years are given the tools to optimise their preconception physical, social and psychological health. Recently, it has also been announced that folic acid will be added to non-wholemeal wheat flour in the UK as a way to

For peer review only - http://bmjopen.bmj.com/site/about/guidelines.xhtml

For peer review only

reduce the incidents of neural tube defects[25]. It is also important to acknowledge that efforts have been made globally. Examples include a policy brief published by the World Health Organization with the aim of improving preconception care[3] and the identification of a condensed set of preconception health indicators in the United States with the aim of better monitoring the health status of women of reproductive age[1].

Currently, no comprehensive synthesis of the evidence relating to this topic is available in the proposed context of the UK and Ireland. Although this review has potential strengths, such as the transparency of the approach adopted, the addition of consultations with experts and the involvement of a PPIE advisory panel, there are also challenges and limitations, such as the broad scope of the review and the lack of a formal critical appraisal of the individual resources. Both strengths and limitations will be clearly explored and further highlighted in the full report.

For peer review only

For peer review only - http://bmjopen.bmj.com/site/about/guidelines.xhtml

References:

1. Robbins CL, D'Angelo D, Zapata L, et al. Preconception health indicators for public health surveillance. *J Womens Health (Larchmt)*2018;27:430-443. doi:10.1089/jwh.2017.6531
2. Public Health England. Making the case for preconception care. 2018.https://www.gov.uk/government/publications/preconception-care-making-the-case (accessed 22 Apr. 2022).
3. World Health Organization. Policy brief: preconception care – maximising the gains for maternal and child health. 2013.https://www.who.int/publications/i/item/WHO-FWC-MCA-13.02 (accessed 22 Apr. 2022).
4. Evans H, Buck D. Tackling multiple unhealthy risk factors. 2018.https://www.kingsfund.org.uk/sites/default/files/2018-03/Tackling%20multiple%20unhealthy%20risk%20factors%20-%20full%20report.pdf (accessed 22 Apr. 2022).
5. Broussard DL, Sappenfield WB, Fussman C, et al. Core state preconception health indicators: a voluntary, multi-state selection process. *Matern Child Health J*2011;15:158-68. doi:10.1007/s10995-010-0575-x
6. Schoenaker DAJM, Stephenson J, Connolly A, et al. Characterising and monitoring preconception health in England: a review of national population-level indicators and core data sources. *J Dev Orig Health Dis*2021;13:137-150. doi:10.1017/S2040174421000258
7. Public Health England. Maternity high impact area: Reducing the inequality of outcomes for women from Black, Asian and Minority Ethnic (BAME) communities and their babies. 2020.https://assets.publishing.service.gov.uk/government/uploads/system/uploads/attachment_data/file/942480/Maternity_high_impact_area_6_Reducing_the_inequality_of_outcomes_for_women_from_Black__Asian_and_Minority_Ethnic__BAME__communities_and_their_babies.pdf (accessed 26 July 2022).
8. Public Health England. Health of women before and during pregnancy: health behaviours, risk factors and inequalities. 2019.https://assets.publishing.service.gov.uk/government/uploads/system/uploads/

For peer review only - http://bmjopen.bmj.com/site/about/guidelines.xhtml

attachment_data/file/844210/Health_of_women_before_and_during_pregnancy_2019.pdf (accessed 22 Apr. 2022).

9. Shawe J, Delbaere I, Ekstrand M, et al. Preconception care policy, guidelines, recommendations and services across six European countries: Belgium (Flanders), Denmark, Italy, the Netherlands, Sweden and the United Kingdom. *Eur J Contracept Reprod Health Care*2015;20:77-87. doi:10.3109/13625187.2014.990088

10. Wellings K, Jones KG, Mercer CH, et al. The prevalence of unplanned pregnancy and associated factors in Britain: findings from the third National Survey of Sexual Attitudes and Lifestyles (Natsal-3). *Lancet*2013;382(9907):1807-1816. Doi: 10.1016/S0140-6736(13)62071-1.

11. Schoenaker DAJM, Stephenson J, Smith H, et al. Women's preconception health in England: a report card based on national maternity services data 2018/19. *Lancet* [Preprint]2022. http://dx.doi.org/10.2139/ssrn.4163702 (accessed 2 Aug. 2022).

12. E, Boyle JA, Walker R, et al. A systematic review of clinical guidelines for preconception care. *Semin Reprod Med*2022;40:157-169. doi:10.1055/s-0042-1748190

13. Arksey H, O'Malley L. Scoping studies: towards a methodological framework. *Int J Soc Res Methodol*2005;8:19-32. doi:10.1080/1364557032000119616

14. Peters MDJ, Marnie C, Tricco AC, et al. Updated methodological guidance for the conduct of scoping reviews. *JBI Evid Synth*2020;18:2119-2126. doi: 10.11124/JBIES-20-00167

15. Tricco AC, Lillie E, Zarin W, et al. PRISMA extension for scoping reviews (PRISMA-ScR): Checklist and Explanation. *Ann Intern Med*2018;169:467-473. doi:10.7326/M18-0850

16. Peters MDJ, Godfrey C, McInerney P, et al. *Chapter 11: Scoping Reviews*. In: Aromataris E, Munn Z (Editors). JBI Manual for evidence synthesis. Adelaide: The Joanna Briggs Institute 2020

17. Gagnon, YC. *The case study as a research method*. Canada: Presses de l'Universite du Quebec 2010.

18. Watson D, Jacob CM, Giles G, et al. A scoping review of nutritional interventions and policy guidelines in the interconception period for prevention of noncommunicable diseases. *Reprod Female Child Health*2022. doi:10.1002/rfc2.8

For peer review only - http://bmjopen.bmj.com/site/about/guidelines.xhtml

19. Pappas C, Williams I. Grey literature: its emerging importance. *J Hosp Librariansh*2011,11:228-234. Doi:10.1080/15323269.2011.587100
20. Aromataris E, Munn Z (Editors). *Chapter 2: Systematic reviews of qualitative evidence.* In: JBI Manual for Evidence Synthesis. *Adelaide:* JBI 2020. https://doi.org/10.46658/JBIMES-20-01
21. Paez A. Grey literature: An important resource in systematic reviews. *J Evid Based Med*. 2017,10:233-240. Doi: 10.1111/jebm.12265.
22. Godin K, Stapleton J, Kirkpatrick SI, et al. Applying systematic review search methods to the grey literature: a case study examining guidelines for school-based breakfast programs in Canada. *Syst Rev*2015;4:138. doi:10.1186/s13643-015-0125-0
23. Department of Health, Social Services and Public Safety. A strategy for maternity care in Northern Ireland. 2012.https://www.health-ni.gov.uk/sites/default/files/publications/dhssps/maternitystrategy.pdf (accessed 5 May 2022).
24. Department of Health. Mental Health Action Plan. 2020.https://www.health-ni.gov.uk/sites/default/files/publications/health/mh-action-plan-plus-covid-response-plan.pdf (accessed 16 February 2023).
25. UK Government. Proposal to add folic acid to flour: consultation response. 2021.https://www.gov.uk/government/consultations/adding-folic-acid-to-flour/outcome/proposal-to-add-folic-acid-to-flour-consultation-response (accessed 20 July 2022).
26. Joosten YA, Israel TL, Williams NA, et al. Community engagement studios: a structured approach to obtaining meaningful input from stakeholders to inform research. *Acad Med*2015;90:1646-50. doi:10.1097/ACM.0000000000000794
27. Staniszewska S, Brett J, Simera I, et al. GRIPP2 reporting checklists: tools to improve reporting of patient and public involvement in research. *Res Involv Engagem*2017;3:13. doi:10.1136/bmj.j3453
28. Sher J. Missed periods. 2016.https://www.nhsggc.org.uk/media/237840/missed-periods-j-sher-may-2016.pdf (accessed 5 May 2022).
29. Department of Health & Social Care. Women's Health Strategy for England. 2022.https://www.gov.uk/government/publications/womens-health-strategy-for-england/womens-health-strategy-for-england (accessed 26 July 2022).

For peer review only - http://bmjopen.bmj.com/site/about/guidelines.xhtml

Authors' Contributions:

EC, LM, MM, LK discussed the protocol and finalised the methodological process of the scoping review. EC drafted the protocol, which was finalised with input from LM, MM, LK, KAE and DS. DT and TS, members of the Patient and Public Involvement and Engagement "Healthy Reproductive Years" panel, also helped shape the research and reviewed the protocol. All authors have seen and approved the final version of the protocol for publication.

Acknowledgements:

The authors would like to acknowledge the contribution of the Patient and Public Involvement and Engagement "Healthy Reproductive Years" panel for their assistance.

Funding Statement:

This work is supported by Queen's University Belfast as part of a PhD studentship for EC. EC is funded by the Department for the Economy NI. DS is supported by the National Institute for Health and Social Care Research (NIHR) Southampton Biomedical Research Centre [IS-BRC-1215-20004]). The views expressed are those of the author(s) and not necessarily those of the NIHR or the Department of Health and Social Care.

Competing Interests Statement:

There is no conflict of interest in this project.

Keywords:

Pre-conception; reproductive health; pregnancy; grey literature.

For peer review only

Appendices:

Appendix I: Preconception risk factors

**Social**		
Adverse childhood experiences<br>Belonging to an ethnic minority<br>Deprivation<br>Domestic abuse (physical and emotional)	Environmental tobacco smoke and second-hand smoke exposure<br>Exposure to toxic or hazardous substances<br>Financial insecurity or unemployment<br>Inadequate support from family or social network	Low educational attainment<br>Migration status<br>Unsuitable access to quality healthcare and dentalcare<br>Unsuitable housing
**Behavioural**		
Alcohol misuse<br>Eating disorders<br>Inadequate dietary habits	Lack of family planning/pregnancy intention<br>Physical inactivity or sedentary behaviours	Smoking<br>Substance misuse<br>Use of teratogenic medications
**Biomedical**		
Fertility issues<br>Folic acid or other vitamin deficiency<br>General health status<br>Genetic risks<br>Infections (e.g., sexually transmitted diseases)	Low immunisation levels<br>Mental health conditions (e.g., anxiety, depression)<br>Parental age<br>Parental obesity or overweight, parental underweight	Physical health conditions (e.g., diabetes mellitus, epilepsy, endometriosis, chronic hypertension)<br>Previous pregnancy and obstetric complications (e.g., previous foetal death, miscarriage, stillbirth, preterm birth)

References for the table: [2,5,6]

For peer review only

Appendix II: Glossary of terms.

Term	Definition
Preconception health	The overall health of non-pregnant individuals of childbearing age[1].
Preconception care	The “services, interventions, support and advice” aimed at optimising pregnancy planning and fitness for pregnancy[2].
Interconception	Period between the end of a pregnancy and the start of the next[29].

For peer review only

For peer review only - http://bmjopen.bmj.com/site/about/guidelines.xhtml

Appendix III: Inclusion and exclusion criteria for a scoping review investigating strategies, policies, services, guidelines, frameworks and recommendations that address preconception health and care for adults in the UK and Ireland.

**Inclusion**	**Exclusion**
The resource is one of the following: strategies, policies, guidelines, frameworks and recommendations, including technical or research reports from government agencies, registered charities or scientific research groups, documents outlining interventions or initiatives from public bodies, articles and guidelines issued by government agencies, Royal Colleges and professional bodies, e-learning resources, leaflets and educational booklets. If the resource is a service, intervention or initiative it will be included only if pertinent to Northern Ireland.	The resource is one of the following: journal articles, preprints (journal articles not yet peer-reviewed or published), working papers from research groups, visual or audio content (or reviews of visual or audio content), academic letters or commentaries, calls for participants, presentations and doctoral dissertations. If the resource is a service, intervention or initiative it will be excluded if not pertinent to Northern Ireland.
The resource addresses preconception health and care.	The resource does not address preconception health and care. This includes if the resource explicitly addresses sexual health, interconception health and care or pregnancy.
The resource is relevant to the UK or Ireland.	The resource is not relevant to the UK or Ireland.
The resource was published, reviewed or updated during or after January 2011.	The resource was published, reviewed or updated before January 2011.
The resource was published in English.	The resource was published in any language other than English.
The resource presents unique insights and does not simply report, cite or signpost other included resources.	The resource mentions preconception health and care only by reporting, citing or signposting included resources, and therefore duplicates findings of an earlier version of the resource.
The resource is currently available.	The resource is not currently available (e.g., guideline being updated).

For peer review only

Appendix VI: Search strategy

List of searches conducted on Advanced Google Search for Northern Ireland.

	**Search terms**
1	~preconception AND "Northern Ireland" OR NI
2	~preconception AND "Northern Ireland" AND policy OR service OR intervention OR guideline OR strategy OR initiative
3	"preconception health" OR "preconception care" AND "Northern Ireland" OR NI
4	~pre-pregnancy AND "Northern Ireland" OR NI AND policy OR service OR intervention OR guideline OR strategy OR initiative
5	~preconception AND "Northern Ireland" OR NI AND ~charity
6	~preconception AND "Health and Social Care Trust" OR HSCT
7	"pregnancy planning" OR "planning a baby" OR "preparation for pregnancy" OR "preparation for parenthood" OR "pregnancy intention" OR "expecting mothers" OR "expectant mothers" OR "conceive" OR "future pregnancy" AND "Northern Ireland"
8	"reproductive health" OR "sexual health" AND "Northern Ireland" AND policy OR service OR intervention OR guideline OR strategy OR initiative

Results limited based on language (English) and country (United Kingdom).

For peer review only - http://bmjopen.bmj.com/site/about/guidelines.xhtml

Appendix V: Additional intended sources and websites

**Source**	**Website**	**Context**
National Institute for Health Research	https://www.nihr.ac.uk/	UK
British National Health Service (NHS)	https://www.nhs.uk/	UK
Royal College of Obstetricians and Gynaecologists	https://www.rcog.org.uk/	UK
Tommy's	https://www.tommys.org/	England, Scotland, Wales
Office for Health Improvement & Disparities	https://www.gov.uk/government/organisations/office-for-health-improvement-and-disparities	England
Public Health Wales	https://phw.nhs.wales/	Wales
Public Health Scotland	https://www.publichealthscotland.scot/	Scotland
NHS Inform	https://www.nhsinform.scot/	Scotland
Health Well	https://healthwell.eani.org.uk	Northern Ireland
Department of Health (Northern Ireland)	https://www.health-ni.gov.uk/	Northern Ireland
Sexual Health NI	https://www.sexualhealthni.info/	Northern Ireland
NI DIRECT	https://www.nidirect.gov.uk/	Northern Ireland
Public Health Agency	https://www.publichealth.hscni.net/	Northern Ireland
Health Service Executive IE	https://www.hse.ie/eng/	Ireland
Health Information and Quality Authority	https://www.hiqa.ie/areas-we-work/health-information/data-collections/all-ireland-public-health-repository	Ireland

For peer review only - http://bmjopen.bmj.com/site/about/guidelines.xhtml

Appendix VI: Example of completed data extraction instrument.

Reference #	Reviewed by	Title & url	Resource format	Source	Date accessed	Date published/last updated or modified	Duration, when applicable	Context	Notes/Short description	Key themes
63	EHC, LM	Hypertension in pregnancy - Quality standard [QS35] > https://www.nice.org.uk/guidance/qs35	Guideline	NICE	02-May-22	2019	NA	UK	"Women of childbearing potential with treated hypertension are given information annually about safe antihypertensive treatment during pregnancy." "Information can be provided to women who may become pregnant about safe antihypertensive treatment during pregnancy as part of an annual review of hypertension care. Women should be informed about potential risks, including the risk of congenital abnormalities, linked to particular antihypertensive drugs. This should enable women to arrange a discussion with the healthcare professional responsible for managing their hypertension about alternative antihypertensive treatments if they are planning pregnancy or become pregnant." (excerpts)	Blood pressure, hypertension, long-term conditions, Preconception advice

For peer review only - http://bmjopen.bmj.com/site/about/guidelines.xhtml

Appendix VII: Revised data extraction instrument.

Data to be extracted	Notes to reviewer
Reference #	
Reviewed by	
Title	
Resource format	e.g., report, intervention, strategy, policy
Source	e.g., Google Advanced Search, NICE, OpenAire
Date accessed	
Year of publication/Year of most recent update.	
Duration	When applicable, the duration of the strategy, policy or intervention will be reported
Participants*	Target population
Context	Country
Target audience*	e.g., healthcare professionals, policymakers
Overarching aim*	e.g., educate, increase awareness
Key notes on topics/content	

* Added following piloting phase.

For peer review only

Preferred Reporting Items for Systematic reviews and Meta-Analyses extension for Scoping Reviews (PRISMA-ScR) Checklist

SECTION	ITEM	PRISMA-ScR CHECKLIST ITEM	REPORTED ON PAGE #
**TITLE**			
Title	1	Identify the report as a scoping review.	i
**ABSTRACT**			
Structured summary	2	Provide a structured summary that includes (as applicable): background, objectives, eligibility criteria, sources of evidence, charting methods, results, and conclusions that relate to the review questions and objectives.	ii
**INTRODUCTION**			
Rationale	3	Describe the rationale for the review in the context of what is already known. Explain why the review questions/objectives lend themselves to a scoping review approach.	1-3
Objectives	4	Provide an explicit statement of the questions and objectives being addressed with reference to their key elements (e.g., population or participants, concepts, and context) or other relevant key elements used to conceptualize the review questions and/or objectives.	3-4
**METHODS**			
Protocol and registration	5	Indicate whether a review protocol exists; state if and where it can be accessed (e.g., a Web address); and if available, provide registration information, including the registration number.	NA
Eligibility criteria	6	Specify characteristics of the sources of evidence used as eligibility criteria (e.g., years considered, language, and publication status), and provide a rationale.	5-6
Information sources*	7	Describe all information sources in the search (e.g., databases with dates of coverage and contact with authors to identify additional sources), as well as the date the most recent search was executed.	7
Search	8	Present the full electronic search strategy for at least 1 database, including any limits used, such that it could be repeated.	Supplementary Material
Selection of sources of evidence†	9	State the process for selecting sources of evidence (i.e., screening and eligibility) included in the scoping review.	8-9
Data charting process‡	10	Describe the methods of charting data from the included sources of evidence (e.g., calibrated forms or forms that have been tested by the team before their use, and whether data charting was done independently or in duplicate) and any processes for obtaining and confirming data from investigators.	9-10
Data items	11	List and define all variables for which data were sought and any assumptions and simplifications made.	NA
Critical appraisal of individual sources of evidence§	12	If done, provide a rationale for conducting a critical appraisal of included sources of evidence; describe the methods used and how this information was used in any data synthesis (if appropriate).	NA
Synthesis of results	13	Describe the methods of handling and summarizing the data that were charted.	9-10

For peer review only - http://bmjopen.bmj.com/site/about/guidelines.xhtml

SECTION	ITEM	PRISMA-ScR CHECKLIST ITEM	REPORTED ON PAGE #
**RESULTS**			
Selection of sources of evidence	14	Give numbers of sources of evidence screened, assessed for eligibility, and included in the review, with reasons for exclusions at each stage, ideally using a flow diagram.	NA
Characteristics of sources of evidence	15	For each source of evidence, present characteristics for which data were charted and provide the citations.	NA
Critical appraisal within sources of evidence	16	If done, present data on critical appraisal of included sources of evidence (see item 12).	NA
Results of individual sources of evidence	17	For each included source of evidence, present the relevant data that were charted that relate to the review questions and objectives.	NA
Synthesis of results	18	Summarize and/or present the charting results as they relate to the review questions and objectives.	NA
**DISCUSSION**			
Summary of evidence	19	Summarize the main results (including an overview of concepts, themes, and types of evidence available), link to the review questions and objectives, and consider the relevance to key groups.	NA
Limitations	20	Discuss the limitations of the scoping review process.	NA
Conclusions	21	Provide a general interpretation of the results with respect to the review questions and objectives, as well as potential implications and/or next steps.	NA
**FUNDING**			
Funding	22	Describe sources of funding for the included sources of evidence, as well as sources of funding for the scoping review. Describe the role of the funders of the scoping review.	16

JBI = Joanna Briggs Institute; PRISMA-ScR = Preferred Reporting Items for Systematic reviews and Meta-Analyses extension for Scoping Reviews.
* Where *sources of evidence* (see second footnote) are compiled from, such as bibliographic databases, social media platforms, and Web sites.
† A more inclusive/heterogeneous term used to account for the different types of evidence or data sources (e.g., quantitative and/or qualitative research, expert opinion, and policy documents) that may be eligible in a scoping review as opposed to only studies. This is not to be confused with *information sources* (see first footnote).
‡ The frameworks by Arksey and O'Malley (6) and Levac and colleagues (7) and the JBI guidance (4, 5) refer to the process of data extraction in a scoping review as data charting.
§ The process of systematically examining research evidence to assess its validity, results, and relevance before using it to inform a decision. This term is used for items 12 and 19 instead of "risk of bias" (which is more applicable to systematic reviews of interventions) to include and acknowledge the various sources of evidence that may be used in a scoping review (e.g., quantitative and/or qualitative research, expert opinion, and policy document).

From: Tricco AC, Lillie E, Zarin W, O'Brien KK, Colquhoun H, Levac D, et al. PRISMA Extension for Scoping Reviews (PRISMAScR): Checklist and Explanation. Ann Intern Med. 2018;169:467–473. doi: 10.7326/M18-0850.

For peer review only - http://bmjopen.bmj.com/site/about/guidelines.xhtml
